# Bidirectional protein–protein interactions control liquid–liquid phase separation of PSD-95 and its interaction partners

**DOI:** 10.1016/j.isci.2022.103808

**Published:** 2022-01-25

**Authors:** Nikolaj Riis Christensen, Christian Parsbæk Pedersen, Vita Sereikaite, Jannik Nedergaard Pedersen, Maria Vistrup-Parry, Andreas Toft Sørensen, Daniel Otzen, Kaare Teilum, Kenneth Lindegaard Madsen, Kristian Strømgaard

**Affiliations:** 1 Center for Biopharmaceuticals, Department of Drug Design and Pharmacology, University of Copenhagen, Universitetsparken 2, 2100 Copenhagen, Denmark; 2 Department of Neuroscience, University of Copenhagen, Blegdamsvej 3B, 2200 Copenhagen, Denmark; 3 Structural Biology and NMR Laboratory & the Linderstrøm-Lang Centre for Protein Science, Department of Biology, University of Copenhagen, Ole Maaløes Vej 5, 2200 Copenhagen, Denmark; 4 Interdisciplinary Nanoscience Center (iNANO), Aarhus University, Gustav Wieds Vej 14, 8000 Aarhus C, Denmark

**Keywords:** Pharmacology, Biomolecules, molecular biology, molecular neuroscience, Biophysics

## Abstract

The organization of the postsynaptic density (PSD), a protein-dense semi-membraneless organelle, is mediated by numerous specific protein–protein interactions (PPIs) which constitute a functional postsynapse. The PSD protein 95 (PSD-95) interacts with a manifold of proteins, including the C-terminal of transmembrane AMPA receptor (AMPAR) regulatory proteins (TARPs). Here, we uncover the minimal essential peptide responsible for the Stargazin (TARP-γ2)-mediated liquid–liquid phase separation (LLPS) formation of PSD-95 and other key protein constituents of the PSD. Furthermore, we find that pharmacological inhibitors of PSD-95 can facilitate the formation of LLPS. We found that in some cases LLPS formation is dependent on multivalent interactions, while in other cases short, highly charged peptides are sufficient to promote LLPS in complex systems. This study offers a new perspective on PSD-95 interactions and their role in LLPS formation, while also considering the role of affinity over multivalency in LLPS systems.

## Highlights

• Charge and valency are important for LLPS formation, both for PSD-95 and the ePSD
• Peptide inhibitors may be used to induce LLPS formation for PSD-95 and the ePSD

## Graphical abstract

**Figure:**
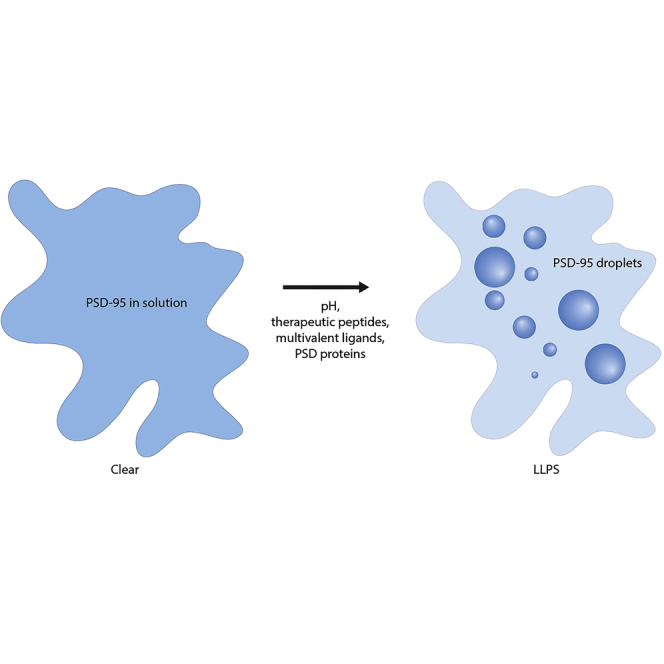

## Introduction

Synaptic transmission is dependent on the proper function and anchoring of ligand-gated ion channels such as the α-amino-3-hydroxy-5-methyl-4-isoxazolepropionic acid receptors (AMPARs), which are responsible for the majority of fast excitatory transmission in the CNS. The postsynaptic density (PSD) contains ∼2000 proteins (Bayes et al., 2012; Bayes and Grant, 2009; Bayes et al., 2011; Distler et al., 2014; Grant, 2019; O'Rourke et al., 2012; Trinidad et al., 2008) and one of the most abundant proteins is PSD protein 95 (PSD-95, 724 residues [Human, Uniprot: P78352], molecular weight 80.5 kDa, with N-terminal palmitoylation 95 kDa) a master scaffold protein of the PSD. PSD-95 regulates the function of AMPARs indirectly through canonical and noncanonical post synaptic density protein (PSD95), Drosophila disc large tumor suppressor (Dlg1), and zonula occludens-1 protein (zo-1) (PDZ) domain-mediated interactions with several members of the TARP family, including Stargazin (Stg) also known as TARP-γ2, thereby anchoring the AMPAR/Stg receptor complex to the PSD membrane and further into the postsynapse (Bissen et al., 2019; Zeng et al., 2019).

PSD-95 typically interact with the C-terminus of a target protein through one of its three PDZ domains, and as for most synaptic PDZ-dependent interactions, the affinities of these interactions are in the micromolar range (Christensen et al., 2019; Stiffler et al., 2006, 2007; Ye et al., 2018; Zhang et al., 2013). Nevertheless, several examples have emerged where PDZ binding is coupled with secondary binding sites, including the lipid membrane, which dramatically potentiates the affinity of the overall interaction (Erlendsson et al., 2019; Janezic et al., 2019; Zeng et al., 2016a, 2016b, 2019). Due to its role as a master scaffold protein in synaptic transmission, PSD-95 has been suggested as a drug target for the treatment of ischemic stroke and chronic pain among others (Ballarin and Tyrmianski, 2018; Hill et al., 2020). Currently, there are a number of lead candidates targeting PSD-95 in both preclinical and clinical development (Christensen et al., 2019), covering both small molecules (Florio et al., 2009; Hu et al., 2013; Lee et al., 2015; Wu et al., 2014) and in particular, peptide-derived compounds (Bach et al., 2009, 2011, 2012; Long et al., 2003; Nissen et al., 2015; Piserchio et al., 2004; Sainlos et al., 2011; Aarts et al., 2002), all of which target the PDZ domains of PSD-95. These molecules feature both monovalent and multivalent interactions with PSD-95, and their affinities range from micromolar (Florio et al., 2009; Piserchio et al., 2004; Aarts et al., 2002) to low nanomolar (Bach et al., 2009, 2012; Nissen et al., 2015).

Recently, liquid–liquid phase separation (LLPS) and the formation of membraneless organelles have emerged as a common feature of protein assembly in many branches of cellular biology (Alberti et al., 2019; Banani et al., 2017). It was recently shown that PSD-95 can undergo LLPS in different ways, both in complex with synaptic Ras GTPase-activating protein (SynGAP) and in complex with additional proteins, such as homer protein homolog 3 (Homer3), SH3, multiple ankyrin repeat domains 3 (Shank3) and guanylate kinase-associated protein (GKAP) (Zeng et al., 2016a). In addition, PSD-95 also undergo LLPS in complex with the Stg C-terminus and the C-termini of other members of the TARP family as well as the C-terminus of N-methyl-d-aspartate receptors (NMDARs) (Tao et al., 2019; Zeng et al., 2016a, 2018, 2019). The LLPS of the key PSD components suggests that the formation of postsynaptic condensates likely govern key aspects of synaptic transmission (Zeng et al., 2019).

In this study, we investigated the behavior of PSD-95 in solution and in complex with multivalent ligands derived from the Stargazin C-terminal region. We show how PSD-95 behaves as a monomeric protein in solution and that PSD-95 can undergo LLPS in the absence and presence of peptide ligands and key protein components of the PSD. We then show how PSD-95 can act as a bidirectional modulator of LLPS formation and confirm a secondary charge dense binding site in Stargazin for PSD-95. Finally, we describe the ability of known, charge-dense, pharmacologically relevant inhibitors of PSD-95 to induce LLPS. Taken together our findings evoke highly charged peptides as potent modulators of synaptic LLPS formation with relevance for the understanding of the plasticity of synaptic efficacy in health and disease and its modulation by peptide therapeutics.

## Results

### Multivalent Stargazin peptides induce liquid–liquid phase separation condensate formation when mixed with postsynaptic density-95

**Figure 1:**
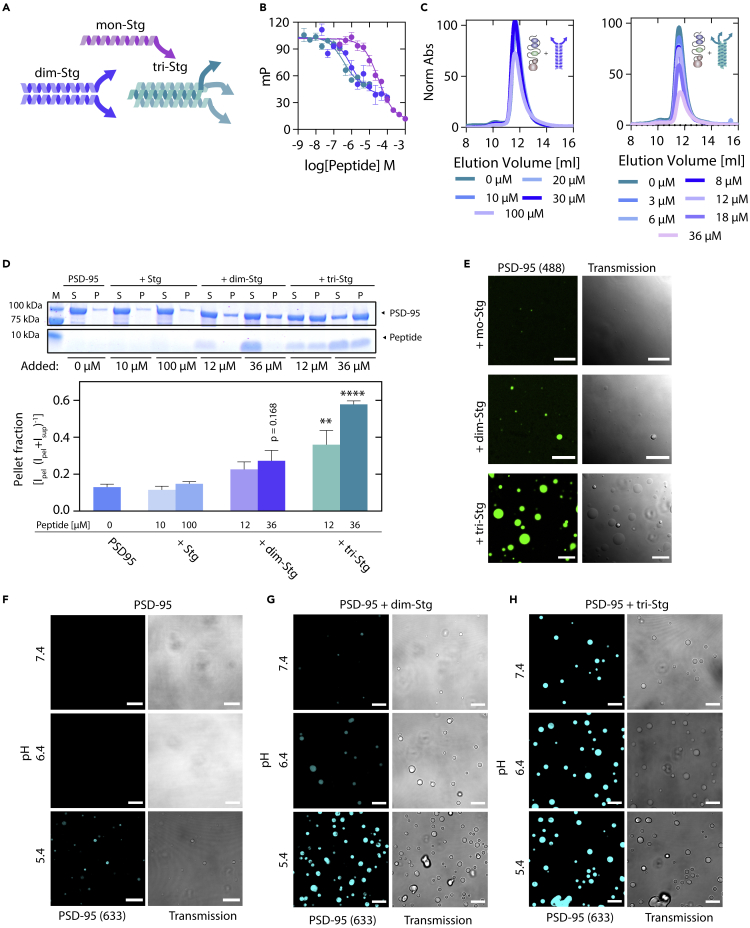
Multivalent PSD-95-peptide interactions can induce concentration and pH-dependent LLPS (A) Illustration of tested monomeric, dimeric, or trimeric peptides. (B) Fluorescence polarization competition with full length PSD-95 shows 33-fold and 81-fold increased affinities for dim-Stg (K_i_ = 237 nM SEM [195; 289] nM, n = 6) and tri-Stg (K_i_ = 98 nM, SEM [81; 119] nM, n = 6) over mono-Stg (K_i_ = 7984 nM, SEM [7146; 8910] nM, n = 6), respectively. (C) Size exclusion chromatography elution profile of PSD-95 (10 μM) incubated with increasing amounts of dim-Stg or tri-Stg. Traces were extracted as absorbance at 280 nm and normalized to the elution of PSD-95 in absence of peptide. (D) SDS-PAGE sedimentation assay with full length PSD-95 incubated with Stg C11, dim-Stg, or tri-Stg indicates the formation of liquid–liquid phase separation (LLPS) condensates for dim-Stg and tri-Stg, but not Stg-C11. (E) LLPS formation was verified for dim-Stg and tri-Stg by confocal microscopy using fluorescently labeled PSD-95 incubated with 36 μM of mono-Stg, dim-Stg, and tri-Stg. Error bars are shown as SEM of (B) n = 6 or (D) n = 3. Statistics was conducted using one-way ANOVA with Dunnett posttest. ∗, p<0.05; ∗∗, p< 0.01; ∗∗∗, p< 0.001; ∗∗∗∗ p<0.0001. (F–H) pH-dependent LLPS formation was seen for PSD-95, in the absence or presence of dim-Stg (G) or tri-Stg (H) using confocal microscopy visualized using fluorescently labeled Alexa 6-PSD-95. Scale bars are 10 μm.

PSD-95 is localized to the PSD by interaction with other proteins such as the AMPAR auxiliary subunit Stg, the GluN2B subunit of the NMDAR, adhesion proteins, including Neuroligns or other scaffolding proteins such as GKAP or SynGAP. Several of the interactions with PSD-95 are multivalent, often due to oligomeric protein assemblies. An example is Stg, which forms a complex with AMPAR, where the Stg:AMPAR stoichiometry can be from 1:1 to 4:1 (Twomey et al., 2016; Zhao et al., 2016). The Stg C-terminal has previously been shown to interact with all the PDZ domains of PSD-95, with a preference for the PDZ1-2 tandem over PDZ3 (Pedersen et al., 2017; Sainlos et al., 2011). To mimic the differences in oligomeric states for the Stg:AMPAR complex, we designed C-terminal Stg peptides which in solution organize as monomers, dimers, or trimers, thereby varying the number of available PDZ-binding motifs (PBMs) targeting PSD-95 between one and 3 (Figure 1A). The dimeric variant was designed using the general control protein GCN4 (GCN4) leucine zipper motif, previously found to form a homo-dimeric parallel helical leucine zipper in solution (Gonzalez et al., 1996), fused to a hexapeptide corresponding to the 6 C-terminal residues of Stg (RRTTPV), through a short flexible linker, yielding a dimeric Stg C6 variant (dim-Stg, Figure 1A). To disrupt the helical GCN4p1 motif, we inserted two prolines, yielding a monomeric conformation of the Stg C-terminal (mono-Stg) (Hanes et al., 1998; Leder et al., 1995). To increase the number of possible PBMs, we made a trimeric GCN4p1 variant (tri-Stg, Figure 1C) (Harbury et al., 1994). As expected, we found that both dim-Stg and tri-Stg displayed a high degree of helicity in solution, with the tri-Stg having higher degree of helicity than dim-Stg, presumably due to more cooperative folding, combined with a lower elution volume for tri-Stg than dim-Stg in size exclusion chromatography, suggesting a larger hydrodynamic radius. The monomer, mono-Stg, displayed a random coil-like structure, and an elution volume similar to dim-Stg (Figures S1A–S1B). Using competitive fluorescence polarization (FP) binding to full length PSD-95 (Zeng et al., 2016b) we found that mono-Stg, dim-Stg, and tri-Stg had apparent affinities of K_i_ = 7984 nM (SEM: [7146; 8910] nM, n = 6), K_i_ = 237 nM (SEM: [195; 289] nM, n = 6) and K_i_ = 98 nM (SEM: [81; 119] nM, n = 6), respectively (Figure 1B). This demonstrates an affinity gain of 33- and 81-fold for dim-Stg and tri-Stg, respectively, over mono-Stg, which is comparable to the 25-fold affinity gain seen for prior work on bivalent Stg peptides (Sainlos et al., 2011).

**Figure 2:**
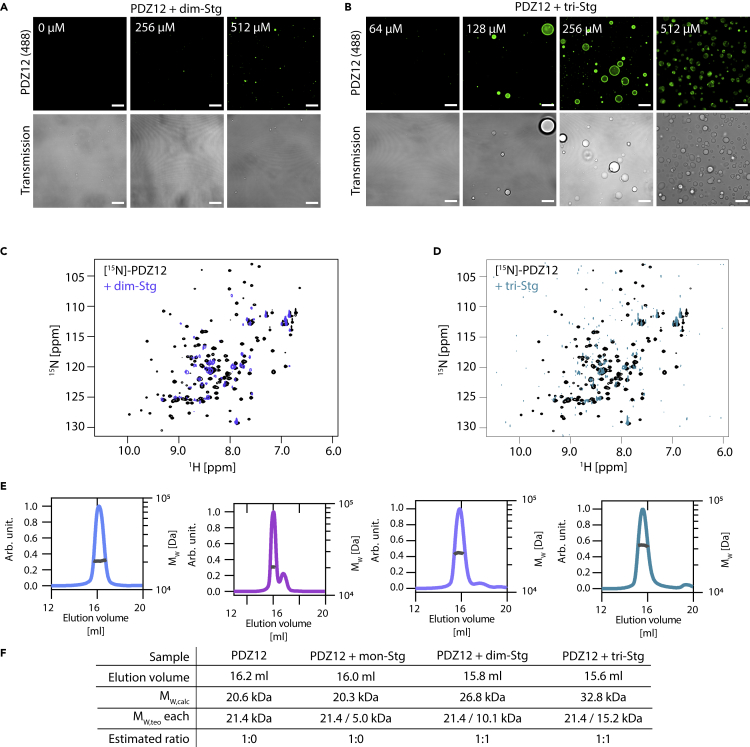
Simple stoichiometric binding of multivalent interaction partners leads to LLPS formation of the PDZ12 tandem from PSD-95 (A) Concentration dependency of LLPS induction for PDZ1-2 incubated with dim-Stg shows only minor LLPS induction. (B) Concentration dependency of LLPS induction for PDZ1-2 incubated with tri-Stg shows LLPS induction at peptide:protein ratios above 1:1 and suggests a biphasic droplet formation. (C and D) [^1^H]-[^15^N]-HSQC spectra overlay of 100 μM [^15^N]-labeled PSD-95 PDZ12 (black) with 512 μM dim-Stg (C) and 512 μM tri-Stg (D), show severe line-broadening likely caused by the dynamic property of the interaction network in the phase-separated droplets. (E) SEC-MALS elution profiles and molecular weight calculation of 200 μM PDZ1-2 (blue) incubated with 600 μM mono-Stg (purple), dim-Stg (blue), or tri-Stg (teal). (F) Data table of fitted data from (E), indicating 1:1 complexes between PDZ1-2 dim-Stg and tri-Stg.Fitting was conducted using ASTRA and data plotting was conducted using GraphPad Prism 8.3. Scale bars are 10 μm.

Size exclusion chromatography (SEC) and SEC multiangle light scattering (SEC-MALS) demonstrated that incubation with either of the peptides did not change the elution volume or molecular weight of PSD-95 (Figures 1C and S1C–S1F). To our surprise, however, we found that an increase in dim-Stg and tri-Stg concentration with a fixed PSD-95 concentration caused a reduction in the total amount of PSD-95/peptide complex eluting from the column (Figure 1C). This was validated using SEC-MALS, also substantiating that no oligomeric PSD-95 eluted from the column (Figures S1C–S1F). Using flow-induced dispersion analysis (FIDA) (Pedersen et al., 2019), we found that the hydrodynamic radius (R_H_) was seemingly larger for the Stg-bound complexes than for PSD-95 in absence of the Stg peptides; however, the R_H_ increase was only significant at 36 μM tri-Stg (∗, p = 0.011, one-way ANOVA) (Figure S1G). Data spikes in the FIDA data also indicated a presence of aggregates, which was not observed in SEC or SEC-MALS, suggesting that the oligomers formed were too large to enter the SEC columns (Figure S1H). We investigated this phenomenon further using an SDS PAGE protein sedimentation assay (Wu et al., 2019; Zeng et al., 2016a, 2018, 2019). We found that both dim-Stg and tri-Stg, but not monomeric Stg, induced a cloudy phase that could be pelleted on centrifugation (Figure 1D). The pellet induction was significant for tri-Stg at 12 μM (∗∗, p< 0.01, one-way ANOVA, Dunnett posttest) and 36 μM (∗∗∗∗, p< 0.0001 one-way ANOVA, Dunnett posttest) (Figure 1D). To evaluate if the Stg C-terminal peptides induced an LLPS transition, we performed fluorescence confocal microscopy of Alexa 488-labeled PSD-95 and unlabeled Stg C-terminal peptides. Indeed, we found that mixing dim-Stg and tri-Stg (at 36 μM) with PSD-95 (3 μM)-induced LLPS droplets (Figure 2E).

LLPS depends on a number of environmental factors including temperature, salt, cosolutes, pH, and the volume excluded by other macromolecules in addition to the concentration of the LLPS forming species itself. Gradients of any of the factors are routinely used to probe LLPS formation and describe the phase diagrams (Alberti et al., 2019). In the context of synaptic function, the LLPS behavior in response to pH changes is particularly interesting, since activity-induced intracellular acidification in neurons of more than one pH unit has been reported to occur in response to seizure-like activity (Raimondo et al., 2012, Siesjö et al., 1985, Xiong et al., 2000), as well as from glutamate receptor activation or membrane depolarization (Hartley and Dubinsky, 1993, Irwin et al., 1994, Svichar et al., 2011, Zhan et al., 1998) . The effect of pH alterations on the protein complex formation in the PSD is currently unknown. We, therefore, investigated the effects of pH on the LLPS assembly of PSD-95 and the Stg C-terminal peptides. Upon acidification to pH 5.4, we observed the formation of a hazy precipitate in the sample tube suggesting sample precipitation (not shown). We note that this effect may be enhanced from a change in the charge of the His-tag used for the purification. Using fluorescence confocal microscopy, we found that the precipitate probed with Alexa 633-labeled PSD-95 behaved as dynamic droplets (Figure 1F), suggesting that PSD-95 can spontaneously undergo LLPS formation in a pH-dependent manner in absence of ligand, also strengthening the notion that PSD-95 can self-organize in larger oligomers in a ligand independent manner. We observed that the formed droplets were dynamic in size and fluorescence recovery after photobleaching (FRAP) experiments consistently demonstrated partial recovery (Figure S1I–S1K). We next tested whether the ability of dim-Stg and tri-Stg and pH works in an additive manner to induce LLPS (Figures 1G and 1H). We found that on the acidification of the buffer there was a pronounced positive effect on LLPS formation for both dim-Stg and tri-Stg (Figures 1G and 1H)., This was also shown by SDS-PAGE sedimentation, where it was evident that tri-Stg enhanced the protein content in the pellet both at pH 6.4 and 5.4 (Figure S2L).

A recent study showed that PSD-95 PDZ1-2 can self-associate into complex structures, (Rodzli et al., 2020), and indeed, we also observed that at high concentrations (1.45 mM) most amide proton resonances in the PDZ1-2 ^1^H-^15^N-HSQC displayed severe line broadening, to an extent where numerous peaks disappeared (Figure S2A). The line broadening was most likely caused by dynamic interactions between individual PSD-95 PDZ1-2 proteins in the intermediate NMR timescale, as these were concentration-dependent (Figure S2A), and the peaks reappeared at a lower concentration. We mapped the concentration-induced intensity changes (Figure S2B) and found that the charged and nonpolar residues for which the intensity is more than 30% reduced or increased, accounted for 82% of the residues, compared to 18% for the polar noncharged residues. Once mapped onto the structure of PSD-95 PDZ1-2 (PDB 3GSL; Figure S2C), we found that all the residues, with intensity changes of more than 30%, were surface exposed and most of the residues were located on the opposite side relative to the binding pocket of both PDZ domains, suggesting that these might be involved in PDZ1-2/PDZ1-2 protein interactions, as have also been suggested from earlier structural work conducted on the PDZ1-2 tandem (Rodzli et al., 2020; Sainlos et al., 2011).

To probe whether the concentration effects seen for PDZ1-2 are similar to the pH-related effects on LLPS of PDZ1-2, induced by lowering the pH, we recorded ^1^H-^15^N-HSQC NMR spectra at pH values of 7.4, 6.4, and 5.4 (Figure S3). As the pH is lowered most of the resonances experience line-broadening, which could indicate the formation of larger species corresponding to the droplets seen by the confocal microscopy (Figures 2 and S2). The pH effects may also be enhanced from a change in the charge of the His-tag used for the purification. Taken together the solution structure of PSD-95 combined with the observation that PSD-95 can undergo LLPS at acidic pH suggests the presence of weak intra-protein interactions both within a single PSD-95 protein and between individual PSD-95 proteins, mediated in part by the PDZ1-2 tandem of PSD-95, which is further accentuated by multivalent ligand binding.

### The PDZ1-2 tandem of postsynaptic density-95 is sufficient to cause liquid–liquid phase separation when mixed with Stargazin peptides

Since PSD-95 is a multi-domain protein, we wanted to evaluate if the PDZ1-2 tandem of PSD-95 could provide a protein scaffold of sufficient valency to promote LLPS. We found that dim-Stg and tri-Stg could induce LLPS of Alexa 488-labeled PDZ1-2 alone, as seen from fluorescence confocal microscopy (Figures 2A and 2B) and SDS-PAGE sedimentation (Figure S1A), similar to the findings for full-length PSD-95. To investigate the residues involved in the LLPS interaction network with dim-Stg and tri-Stg, we recorded ^1^H-^15^N-HSQC NMR spectra of PSD-95 in complex with the two peptides (Figures 2C and 2D). The majority of resonances of the two samples experienced severe line-broadening compared to the absence of the peptides, which is likely caused by the dynamic properties of the interaction networks in the phase-separated droplets. Interestingly, the concentrations required to induce LLPS are higher for the PDZ1-2 than for the full-length protein. This might suggest that other regions of PSD-95, such as PDZ3, in addition to PDZ1-2, also contribute to PSD-95-mediated LLPS formation.

We used SEC-MALS to estimate the binding valency and found that both the elution volume and the molecular weight of the eluting complex are consistent with a 1:1 stoichiometry of the interaction between PDZ1-2 and the Stg peptides at a concentration below the critical LLPS concentration (Figures 2E and 2F). Taken together these data support that the tandem PDZ1-2 protein combined with dim-Stg or tri-Stg is sufficient for LLPS formation and suggest that the LLPS core of PSD-95 is the PDZ1-2 tandem.

### Postsynaptic density-95 serves as a reversible, negative modulator of condensate formation governed by multivalent PDZ interactions

**Figure 3:**
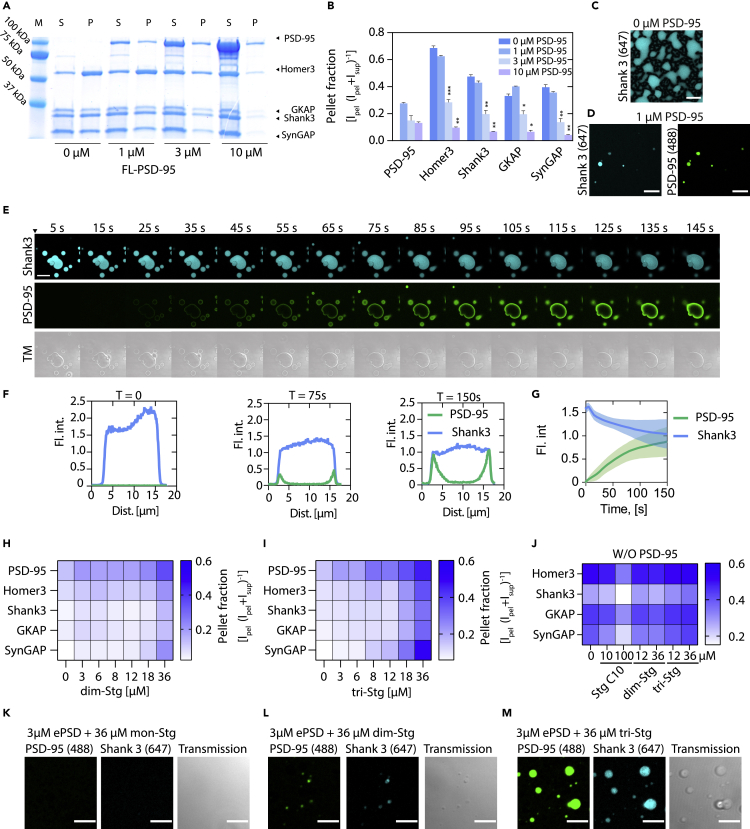
ePSD condensate can be modulated through multivalent PSD-95 PDZ interactions (A) Representative SDS-PAGE gel of sedimentation assay with 3 μM Homer3, Shank3, GKAP, and SynGAP (H-S-G-S) incubated with increasing amounts of PSD-95. (B) Quantification of the gels shown in (A) shows the reduction of condensate formation as a function of PSD-95 addition. Statistics was conducted using one-way ANOVA with Dunnett posttest. ∗, p<0.05; ∗∗, p< 0.01; ∗∗∗, p<0.001; ∗∗∗∗ p< 0.0001. (C) Validation of H-S-G-S LLPS condensate formation using confocal microscopy, with fluorescently labeled Shank3, scale bars 10 μm. (D) Confocal microscopy of 5x ePSD condensate with fluorescently labeled PSD-95 and Shank3, scale bar 10 μm. (E) Confocal microscopy time series of H-S-G-S condensate upon the addition of PSD-95 indicates the slow absorption of PSD-95 into existing droplets. (F) Line intensity profile of PSD-95 (Green) and Shank3 (blue) at indicated timepoints, which show a time-dependent reduction in Shank3 signal. (G) Quantification of mean droplet intensity of Shank3 (blue) and PSD-95 (green) after the addition of PSD-95. Error band is the SEM of three independent chambers. (H and I) Heatmap of SDS-PAGE sedimentation quantification of 5xePSD (3 μM H-S-G-S, 10 μM PSD-95) incubated with increasing amounts of dim-Stg (H) or tri-Stg (I). (J) Heatmap representation of SDS-PAGE sedimentation quantification of H-S-G-S condensate (3 μM H-S-G-S) incubated with Stg C10, dim-Stg, or tri-Stg. (K-M) confocal microscopy confirmation of LLPS formation on the addition of dim-Stg and tri-Stg (36 μM) to ePSD (3 μM). Error bars are shown as SEM of n = 3, scale bars 5 μm.

Based on earlier observations on the mutual impact of five major synaptic scaffold proteins (PSD-95, Homer3, Shank3, GKAP, and SynGAP, *see Methods*) (Zeng et al., 2016a, 2018), and the formation of LLPS droplets on mixing (Zeng et al., 2016a, 2018, 2019), we wanted to explore this further and therefore, expressed and purified these five major synaptic scaffold proteins. We found that, in the absence of PSD-95, the complex composed of Homer3, Shank3, GKAP, and SynGAP (H-S-G-S) (3 μM each), condensed into LLPS droplets (Figure 3A). Surprisingly, on incubation with increasing concentrations of PSD-95, this phase separation was significantly reduced in presence of PSD-95 (3 μM) (∗∗∗ for Homer3, p<0.001; ∗∗ for Shank3, p< 0.01; ∗ for GKAP p = 0.0113; ∗∗ for SynGAP, p< 0.01; two-way ANOVA Dunnett posttest) and at 10 μM of PSD-95 (∗∗ for Homer3, p< 0.01; ∗∗ for Shank3, p< 0.01; ∗ for GKAP p = 0.013; ∗∗ for SynGAP, p< 0.01; two-way ANOVA Dunnett posttest) (Figure 3B). This was confirmed using confocal microscopy, where we observed LLPS droplets formed by 3 μM of H-S-G-S in the absence of PSD-95 (Figure 3C) while only minor droplets were seen in the presence of PSD-95 (1 μM) (Figure 3D). To visualize the negative modulation of the LLPS of the H-S-G-S-complex PSD-95 (8 μM) was added to pre-existing H-S-G-S condensates (3 μM each), probed with Alexa 647-labeled Shank3 (0.3 μM) and Alexa 488-labeled PSD-95 (0.8 μM) (Figure 3E). When PSD-95 associated with the droplets, there was slow incorporation of PSD-95 into the droplets from the periphery that gradually reduced Shank3 intensity (Figure 3F), which was quantified to ∼30% reduction in Shank3 intensity (Figure 3G). No effect was seen on the addition of PBS (Figures S4A–S4B) indicating a PSD-95-dependent disassembly of the H-S-G-S condensate.

We next examined how the di- and trimeric Stg peptides would affect the destabilized condensate of the five major excitatory postsynaptic density (ePSD) proteins. Indeed, condensate formation was facilitated with increasing concentration of dim-Stg and, in particular, tri-Stg (Figures 3H–3I and S5A–S5D), but not mono-Stg (Figures S4C and S5E–S5F), suggesting that the peptide valency and concentration are critical for ePSD condensate stabilization. The induction of LLPS in presence of dim-Stg and tri-Stg was validated by confocal microscopy, which suggested that tri-Stg was much more effective than mono-Stg and dim-Stg at stabilizing LLPS in the ePSD (Figures 3K–3M). While dim-Stg and tri-Stg could be used to induce LLPS in the ePSD complex, the H-S-G-S condensates were unaffected by dim-Stg and tri-Stg, while high concentrations of mono-Stg seemed to reduce the pelleting of the H-S-G-S condensate (Figures 3J and S5G–S5H).

These data demonstrate a critical role of PSD-95 in the negative modulation of the ePSD condensates, which in turn is governed by specific multivalent PDZ domain interactions.

### Stargazin contains multiple postsynaptic density-95 binding sites

**Figure 4:**
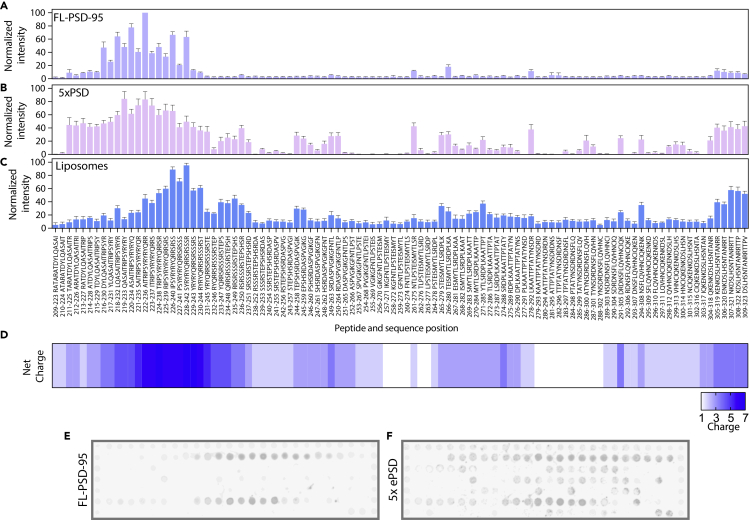
PSD-95 and ePSD condensate relies on a binding site outside the PDZ motif (A–C) Quantification of CelluSPOT arrays of Stg C-terminal peptides (16-mers) when incubated with PSD-95 (A), ePSD (B), or vesicles (C). Primary sequence of peptides is indicated below each bar. Error bars are shown as SEM of n = 8 for PSD-95 binding, n = 6 for ePSD and n = 8 for vesicles. (D) Heatmap of peptide net charge. (E and F) Representative celluSPOT array membranes of Stg C-terminal as 16-mer peptides, with a single residue frameshift per peptide, when incubated with PSD-95 (1 μM, (E)) or ePSD (3 μM H-S-G-S and 1 μM PSD-95, (F)).

It was recently shown that the full-length Stg C-terminus can induce LLPS when mixed with PSD-95 alone or in combination with Homer3, Shank3, GKAP, and SynGAP (Zeng et al., 2019). This effect is repressed by S-to-D mutations in an S/R-rich region (S221-S253, Uniprot: Q9Y698) of the Stg C-terminus positioned in the membrane-proximal region upstream of the PBM (T321-V323) (Feng et al., 2019b; Zeng et al., 2019). The Stg C-terminus (Figure S6A) was shown to interact with PDZ2 through the PBM and noncanonically with PDZ1 via the S/R-rich region probably interacting with E/D residues positioned opposite to the canonical PDZ1 binding pocket in PDZ1 (Zeng et al., 2019). To characterize the non-canonical PDZ interaction of PSD-95 with the Stg C-terminus, we designed and prepared a celluSPOT peptide array (Winkler et al., 2009; Wu and Li, 2009) of the entire Stg C-terminus consisting of 101 15-mer peptides (Figure 4A), consecutively shifting one residue toward the C-terminus. To reduce nonspecific binding to the celluSPOT membrane, the membrane was blocked by incubation with BSA before incubation with the protein. The celluSPOT approach involves the coupling of the C-terminal carboxylic acid to the cellulose membrane, thereby blocking canonical PDZ interactions and probing only noncanonical PDZ interactions of the Stg C-terminus with PSD-95. In addition, we included 39 peptides, carrying the S-to-D mutations in the S/R-rich region described earlier (Sumioka et al., 2010; Tomita et al., 2005), to address putative modulation by phosphorylation.

We found that the fluorescence intensities in the membrane-proximal region of Stg (A214-E245), which overlaps partially with the charged S/R-rich region (Figure 4A), were selectively enhanced on the addition of PSD-95 (1 μM total, 0.9 μM unlabeled PSD-95 and 0.1 μM Alexa 633-labeled PSD-95), suggesting a secondary binding site for PSD-95 in the A214-E245 region (Figures 4A and S6B). The peptide array further suggested that the S-to-D variants (Figure S6C) slightly reduced the binding to isolated PSD-95 in the membrane-proximal region, confining the binding region from residues T211-S253 to T215-D241 (Figure S6D). To validate the binding of the S/R-rich region of Stg to PSD-95, we then performed FP on a fluorescently labeled 15-mer peptide (TAMRA-G-A_222_ITRIPSYRYRYQRR_236_) representing the core binding region and found that PSD-95, indeed, bind to Stg_A222-R236_ (K_d_ = 14.9 ± 3.0 μM) (Figure S6E). Interestingly, the S/R-rich region overlaps with the binding site of Arc (Zhang et al., 2015), which also shows binding to Stg_A222-R236_ (K_d_ = 8.37 ± 1.25 μM) (Figures S7A–S7B).

Next, adding the other four proteins of the ePSD (1 μM of H-S-G-S and 0.9 μM unlabeled PSD-95 and 0.1 μM Alexa 633-labeled PSD-95) to the Stg array caused a potentiation and broadening of the PSD-95 signal now covering the T211-S253 region as well as at the extreme C-terminus (Q304-V323) of Stg (Figures 4B and S8). All ePSD proteins were also tested individually for binding to the Stg C-terminal peptides, which resulted in a measurable intensity in the same membrane-proximal region for all proteins (Figure S9A), suggesting a low specificity interaction, which was supported by FP binding data (Figure S9B). Moreover, the negative effect of the S-to-D mutations on PSD-95 binding was maintained in presence of the ePSD complex, in particular in the R225-A252 region (Figure S9C).

The Stg C-terminus has previously been shown to interact also with lipid membranes in an S-to-D-dependent manner (Sumioka et al., 2010). We, therefore, tested binding of liposomes made from bovine brain lipids, to the array and found that the area Q219-D251, partially overlapping with the protein binding region, as well as the C-terminal region Q304-V323 indicated liposome binding (Figure 4C), in accordance with a previously reported lipid-binding site of the Stg C-terminal (Sumioka et al., 2010). This binding largely overlaps with the overall charge distribution of the peptides (Figure S9E). Accordingly, the S-to-D mutations reduced the binding of the liposomes in this area (Figure S6D and S9C–S9D), suggesting that the charge of the peptides is important for the binding of the liposomes.

Taken together, the peptide array and binding experiments suggest that a secondary PSD-95 binding site in the Stg C-terminus is confined to the region A214-E245, which largely overlaps with the lipid-binding site covering the region Q219-D251 (Sumioka et al., 2010) and is consistent with the suggestion that the S/R-rich region is also involved in the binding of PSD-95 in a way which enhances affinity (Zeng et al., 2019).

### Stg_A222-R236_ can induce liquid–liquid phase separation

**Figure 5:**
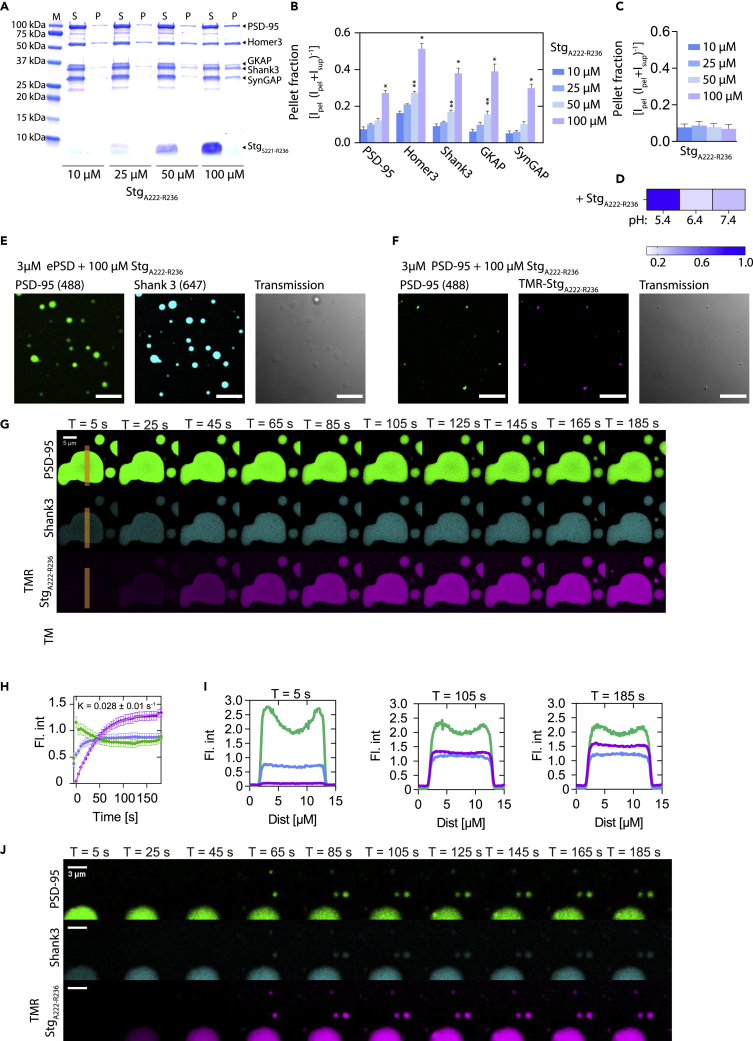
Stg_A222-R236_ is sufficient to induce LLPS. (A–B) SDS-PAGE sedimentation assay with ePSD (3 μM) incubated with increasing amounts of Stg_A222-R236_, show increasing LLPS (A) SDS-PAGE sedimentation assay with 5xePSD (3 μM) incubated with increasing amounts of Stg_A222-R236_. (B) Quantification of the gels shown in (A) shows increased tendency for LLPS formation with increasing concentrations of Stg_A222-R236._ Statistics was conducted using one-way ANOVA with Dunnett posttest. ∗, p<0.05; ∗∗, p< 0.01; ∗∗∗, p< 0.001; ∗∗∗∗ p< 0.0001. (C) Quantification of SDS-PAGE sedimentation assay with PSD-95 (10 μM) incubated with increasing amounts of Stg_A222-R236_. (D) pH titration of PSD-95 in presence of Stg_A222-R236_ shows increased LLPS formation at lower pH. (E and F) Images validating that Stg_A222-R236_ can induce LLPS for the ePSD (D) but not for PSD-95 (E) alone. Scale bars indicate 10 μm. (G) Time series of H-S-G-S condensate first incubated with PSD-95 and subsequent addition of TMR labeled Stg_A222-R236_, shows uptake into existing droplets. Scale bars indicate 5 μm. (H) Quantification of mean droplet fluorescence intensity shows rapid uptake of TMR Stg_A222-R236_. Error bars are shown as SEM of 17 droplets, fitting was conducted using GraphPad Prism using a single exponential association fit. (I) Line scans of droplet intensity for PSD-95 (green), Shank (blue), and Stg_A222-R236_ (purple). (J) Time series of H-S-G-S condensate first incubated with PSD-95 and subsequent addition of TMR labeled Stg_A222-R236_, shows the formation of new droplets. Scale bars indicate 3 μm.

**Figure 6:**
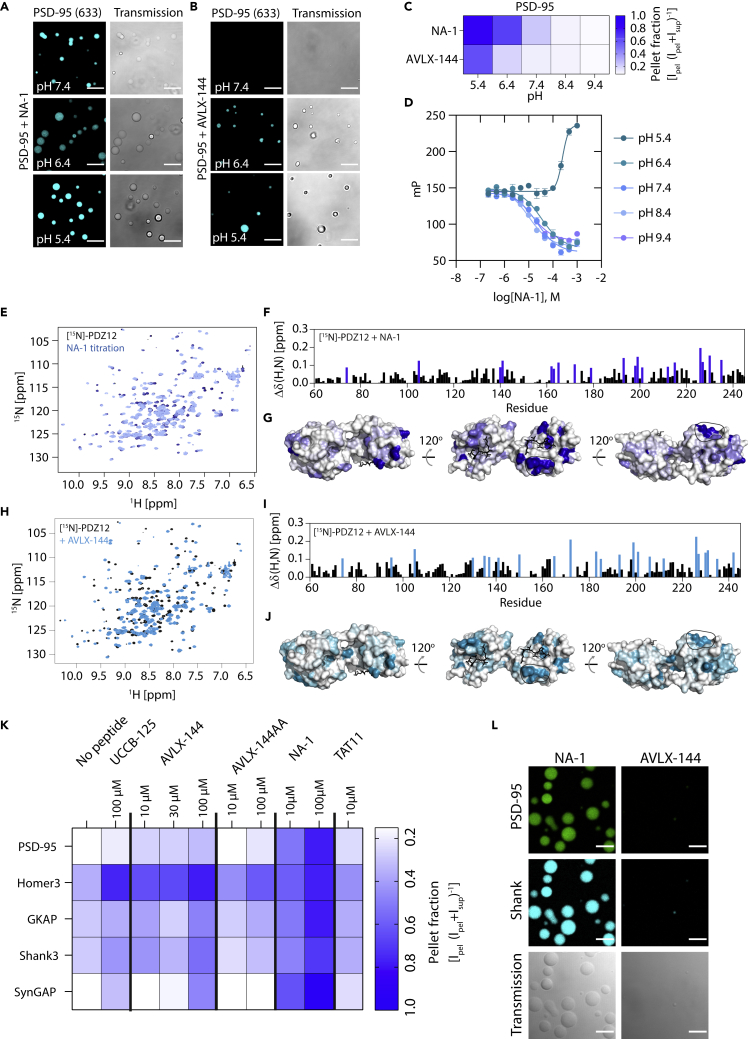
Pharmacological inhibitors of PSD-95 affect LLPS formation for PSD-95 and ePSD (A and B) Confocal microscopy shows that NA-1 (A) and AVLX-144 (B) and induces LLPS once complex with PSD-95 at indicated pH values. (C) Heatmap representation of SDS-PAGE sedimentation quantification of NA-1 and AVLX-144 pH-dependent LLPS induction. Scale bars indicate 10 μm. (D) Competitive FP at different pHs suggests drastic changes in the complex size at low pH, as indicated by the upward sigmoidal curve, compared to the downward sigmoidal curves for higher pH values. (E) [^1^H]-[^15^N]-HSQC spectra overlay of 100 M PDZ1-2 titrated with NA-1 peptide concentrations ranging from 4 μM to 500 μM. (F) Chemical shift perturbation on PDZ1-2 (PDB: 3GSL) upon the addition of NA-1 shows wide-spread perturbations in the PDZ1-2 tandem. Blue bars indicate perturbations larger than the mean Δδ(H,N) + 1 Std. Dev. (G) Surface representation of PDZ1-2 with NA-1 inducted perturbations larger than the mean Δδ(H,N) + 1 Std. Dev. Highlighted in blue, while remaining Δδ(H,N) were colored with a gradient from blue to white according to their Δδ(H,N). The black ligands represent RTTPV, which was docked into the PDZ binding pocket of both PDZ1 and PDZ2 using alignment to PDB ID 3JXT (Sainlos et al., 2011). (H) [^1^H]-[^15^N]-HSQC spectra overlay of free 100 μM PDZ1-2 (black) and with 512 μM AVLX-144 peptide and PDZ1-2 (teal). (I) Chemical shift perturbation on PDZ1-2 on the addition of AVLX-144 shows widespread perturbations in the PDZ1-2 tandem. Teal bars indicate perturbations larger than the mean Δδ(H,N) + 1 Std. Dev. (J) Surface representation of PDZ1-2 (PDB: 3GSL) with AVLX-144 induced perturbations larger than the mean Δδ(H,N) + 1 Std. Dev. Highlighted as teal, while remaining Δδ(H,N) were colored with a gradient from teal to white according to their Δδ(H,N). The black ligands represent RTTPV, which was docked into the PDZ binding pocket of both PDZ1 and PDZ2 using alignment to PDB ID 3JXT. (K) Heatmap representation of SDS-PAGE sedimentation quantification of indicated peptides (see Figure S12 gels and quantification). (L) Confocal microscopy shows that NA-1, but not AVLX-144, induces LLPS once complexed with the ePSD. Scale bars indicate 10 μm.

As the Stg C-terminal was recently shown to induce LLPS in an Arg (R) and Ser-to-Asp (S-to-D)-dependent manner, where the substitution of specific R or S residues in the Stg C-terminal (S/R-rich region) disrupted the LLPS formation (Zeng et al., 2019), we wondered whether the 15-mer Stg_A222-R236_ peptide, which is part of the S/R-rich region, was sufficient to induce LLPS. Indeed, we found that the Stg_A222-R236_ peptide (100 μM) induced LLPS for the ePSD (3 μM) (Figures 5A, 5B, and 6E). However, Stg_A222-R236_ (100 μM) did not induce LLPS for PSD-95 alone (3 μM) at pH 7.4 but LLPS was observed at the slightly more acidic pH 5.4 (Figures 5D and S10B), similar to what was observed for PSD-95 in absence of ligands. As demonstrated above, the addition of PSD-95 to existing H-S-G-S droplets resulted in a reduction in Shank3 intensity, while PSD-95 was slowly taken up into the droplets from the periphery of pre-existing droplets (Figure 3E). When adding Stg_A222-R236_ to H-S-G-S droplets, which had first been subjected to the addition of PSD-95, we found that Stg_A222-R236_ was taken up rapidly by existing droplets (Figure 5E) with an almost constant intensity profile across the droplets (Figures 5G–5I), as opposed to the peripheral localization seen for PSD-95 (Figures 3E–3G). Further, the peripheral localization for PSD-95 was reversed in presence of Stg_A222-R236_ droplets, suggesting alterations in the dynamic protein network (Figures 5G–5I).

In addition to the integration into existing droplets, Stg_A222-R236_ increased the LLPS formation of the ePSD as evidenced by the appearance of new, smaller, droplets over the time of the experiment (Figure 5J).

The above sedimentation assays and imaging demonstrated that a 15-mer peptide comprising residues A222-R236 derived from the Stg C-terminal, while inadequate for the formation of LLPS with PSD-95 alone, is sufficient to promote LLPS condensate formation for the ePSD and to reorganize the dynamic network.

### Known inhibitors of the postsynaptic density-95 PDZ domains can influence liquid–liquid phase separation

Several inhibitors, of different valencies, similarly to the different Stg variants presented earlier herein, have been developed to target the PDZ domains of PSD-95, including two promising drug candidates that further feature Arg-rich cell-penetrating peptides (CPPs) (Bach et al., 2012; Christensen et al., 2019; Aarts et al., 2002). This includes the monomeric peptide NA-1 (nerinetide, Aarts et al., 2002) and the bivalent peptide derivative AVLX-144 (Bach et al., 2012). As we found that both valency and positively charged residues induce LLPS, we wanted to investigate if this was also the case for clinically relevant drug candidates, targeting PSD-95. Thus, we wanted to investigate whether the two peptide inhibitors of PSD-95, NA-1, and AVLX-144, affected LLPS formation for either PSD-95 or the ePSD. NA-1 (Aarts et al., 2002) is a monomeric 20-mer peptide containing the 9 C-terminal amino acids from the GluN2B NMDAR subunit, fused to an N-terminal 11-mer peptide from the *trans*-activator of transcription protein (TAT), which facilitates the membrane penetration of NA-1. The clinical investigations of NA-1 have provided the compound in a dosage of 2.6 mg/kg, which corresponds approximately to 1 μM the compound distributed in the entire body volume. AVLX-144 (Bach et al., 2012) is likewise, derived from the GluN2B C-terminal, but is a bivalent inhibitor, also comprising a TAT peptide. Both NA-1 and AVLX-144 target the first two PDZ domains of PSD-95 through canonical PDZ interactions (Bach et al., 2012; Aarts et al., 2002) and both adopt a random coil structure in solution (Figure S12A).

We found that NA-1 (36 μM) induced LLPS when incubated with Alexa 633-PSD-95 (30 nM labeled/3 μM unlabeled) at pH below 7.4 (Figure 6A), while AVLX-144 only induced LLPS at pH below 6.4 (Figure 6B). This was validated using SDS-PAGE sedimentation (Figures 6C, S10B and S10C). To show that the pH change did not compromise the affinity of NA-1 toward PSD-95, we did competitive FP binding. At pH 5.4, we saw an inverted competition curve (Figure 6D), suggesting the formation of larger molecular assemblies as a function of NA-1 concentration (K_i,app, pH 5.4_ = 156 μM, SEM interval: [155–158] μM). This suggests that NA-1 can induce LLPS at a more acidic pH of pH 5.4, but importantly also at very low PSD-95 concentrations ([C_PSD-95_] = 150 nM). Interestingly, we observed only minor changes in K_i_ as a function of pH (Figure 6D), in the pH range 6.4–9.4 (K_i,app, pH 6.4_ = 7.2 μM, SEM interval: [6.0–8.4] μM; K_i,app, pH 7.4_ = 4.5 μM, SEM interval: [3.4–5.7] μM; K_i,app, pH 8.4_ = 2.6 μM, SEM interval: [1.5–3.8] μM; K_i,app, pH 9.4_ = 4.1 μM, SEM interval: [2.9–5.2] μM), this was also the case for the saturation binding of 5FAM-labeled AVLX-144 (Figure S11D).

To obtain molecular insight into the differential propensity of NA-1 and AVLX-144 to cause LPPS, we recorded ^1^H-^15^N-HSQC NMR spectra of PDZ1-2 with and without these peptides (Figures 6E–6J). Both peptides caused similar changes of the chemical shifts throughout the primary sequence of PDZ1-2 suggesting comparable binding for both NA-1 and AVLX-144. Both NA-1 and AVLX-144 perturbed residues in PDZ2 to a similar extent, while the perturbations in PDZ1 were stronger for AVLX-144 (Figures 6F and 6G) than for NA-1 (Figures 6I and 6J), in concordance with dual occupancy of both PDZ domains in the PDZ1-2 tandem for AVLX-144 (Bach et al., 2012; Chi et al., 2010; Aarts et al., 2002). We suspected that the positively charged TAT peptide in NA-1 was able to utilize the negatively charged patch on PDZ1, as have been suggested to be the secondary binding site for the S/R-rich region of Stg (Zeng et al., 2019), but we did not see any perturbations indicating this, for neither NA-1 nor AVLX-144. We also tested the 11-mer TAT peptide alone and Stg_A222-R236_, which did not result in any chemical shift perturbations in the recorded ^1^H-^15^N-HSQCs of PSD-95 (512 μM Stg_A222-R236_/128 μM ^15^N-PDZ1-2). The reason for the lack of perturbations might be that the isolated PDZ1 interactions, as presented earlier, occur on a timescale that is outside of the experimental window.

### NA-1 can induce liquid–liquid phase separation condensate formation in the excitatory PSD complex

Expanding these findings into the excitatory PSD (ePSD) system, we found that NA-1 could induce LLPS at 10 μM (∗ for PSD-95, p< 0.0205; ns for Homer3, p = 0.0947; ns for Shank3, p = 0.0872; ∗ for GKAP p = 0.0252; ∗ for SynGAP, p = 0.0286; two-way ANOVA Dunnett posttest) and 100 μM (∗∗∗ for PSD-95, p< 0.001; ∗∗ for Homer3, p< 0.01; ∗ for Shank3, p = 0.0280; ∗∗ for GKAP p< 0.01; ∗∗ for SynGAP, p< 0.01; two-way ANOVA Dunnett posttest) in the PSD-95 containing ePSD (Figures 6K and S12). On the other hand, AVLX-144 induced LLPS to a minor extent (Figures 6E and S12) at 10 μM (∗ for PSD, p = 0.0190; ns for Homer3, p = 0.185; ns for Shank3, p = 0.910; ns for GKAP, p = 0.993; ns for SynGAP, p = 0.148; two-way ANOVA Dunnett posttest) and 100 μM (∗ for PSD, p = 0.0357; ns for Homer3, p = 0.117; ns for Shank3, p = 0.814; ns for GKAP, p = 0.993; ns for SynGAP, p = 0.247; two-way ANOVA Dunnett posttest). Mutation of the PBM of AVLX-144 (TDV to ADA) to generate AVLX-144-AA, a nonbinding version, largely compromised the ability to drive LLPS of the ePSD (Figure S12). The TAT peptide alone (10 μM) induced LLPS (∗∗ for PSD-95, p< 0.01; ns for Homer3, p = 0.05; ∗ for Shank3, p = 0.0189; ∗ for GKAP p = 0.0252; ns for SynGAP, p = 0.0949; two-way ANOVA Dunnett posttest) of the ePSD similar to that of AVLX-144 (Figures 6K and S12). Removal of the TAT peptide from AVLX-144, generating AVLX-125 (UCCB-125), did not alter the ePSD LLPS formation (Figure 6K). AVLX-125 (100 μM) pellet formation was only significant for Homer3 and SynGAP (ns for PSD-95, p = 0.45; ∗ for Homer3, p< 0.05; ns for Shank3, p = 0.87; ns for GKAP p> 0.99; ∗∗ for SynGAP, p = 0.0026; two-way ANOVA Dunnett posttest) of the ePSD similar to AVLX-144 (Figures 7K and S12G). Taken together these data suggest that both charge and valency is important for LLPS formation, both for PSD-95 and the ePSD. The data also suggest a potential new aspect on peptide-mediated inhibition of PPIs, proposing that peptide-based inhibitors may be designed for and act to modulate LLPS of PPI networks, thus suggesting a novel way of targeting protein–protein interactions.

## Discussion

The recent discovery of phase separation of key PSD components including PSD-95 provides a new paradigm for the understanding of synaptic biology but also highlights the complexity of these phenomena. Since it was shown that proteins greatly concentrate in the condensate droplets, LLPS has emerged as a possible explanation of how the postsynapse can be immensely enriched in proteins, and how these are sorted on the basis of their protein interaction networks (Alberti et al., 2019; Feng et al., 2019a; Yoshizawa et al., 2020; Zeng et al., 2016a, 2018, 2019).

As shown recently PDZ1-2 can undergo spontaneous oligomerization (Rodzli et al., 2020), a fact that we also observed when increasing the PDZ1-2 concentration to the millimolar range. Under normal circumstances, protein concentrations in the millimolar range are considered nonphysiological, but due to recent developments in cell biology, mainly focused on LLPS (Feng et al., 2019a, 2019b; Yoshizawa et al., 2020), it seems increasingly common that protein complexes which undergo LLPS are of high micromolar and even millimolar concentration in the LLPS droplets (Feng et al., 2019a, 2019b; Yoshizawa et al., 2020), suggesting that even low-affinity and low-specificity interactions become of importance in these supracomplexes.

The ability of PSD-95 to engage in LLPS has recently been described in great detail in pioneering work by Zhang and coworkers (Feng et al., 2019b; Tao et al., 2019; Zeng et al., 2016a, 2019). Here, we observed that Shank3, Homer3, GKAP, and SynGAP together undergo LLPS at low concentrations and that PSD-95 acts as a negative regulator for LLPS when mixed with the remaining components. This occurs in absence of a PSD-95 PDZ1-2 specific ligand. PSD-95 has earlier been shown to facilitate LLPS in the ePSD system, but in earlier cases, this negative regulatory effect has not been observed. Of note, SynGAP and Stg PDZ-binding motifs have been suggested to interact with all PDZ domains of PSD-95 (Hafner et al., 2015, Walkup et al., 2016), thus, they may well compete for the same PDZ domains on PSD-95, potentially further modulating LLPS formation in synapses.

Earlier work conducted on the ePSD system (Zeng et al., 2018, 2019) shows that PSD-95 does not actively participate in LLPS in the absence of a multivalent ligand, a tetrameric construct of the GluN2B C-terminal, also using the GCN4p1 backbone (Zeng et al., 2018); however, based on our experiments using the multivalent Stg constructs, and previous publications (Zeng et al., 2016a, 2018, 2019), it is evident that PSD-95 can undergo separate LLPS, which subsequently can incorporate into the Homer/Shank/GKAP LLPS system, through low-affinity GKAP linkage (K_D_ = 176 μM) (Zeng et al., 2018)).

It is known that low and moderate affinity interactions can drive systems toward LLPS, as is the case here for both the isolated Stg_A222-R236_ peptide and the PSD-95 inhibitor, NA-1. This has also been shown earlier for intrinsically disordered proteins, which in some cases can act as promiscuous LLPS drivers (Protter et al., 2018) and Arg-rich peptides can induce LLPS in large protein sets (Boeynaems et al., 2017).

NA-1 and AVLX-144 are both promising clinical drug candidates in the treatment of acute ischemic stroke (Bach et al., 2019; Hill et al., 2020). In our experiments, we were able to demonstrate that NA-1 induced LLPS on interaction with isolated PSD-95 and also in the more complex setting of the ePSD, in which case the concentration of NA-1, which induces LLPS, is close to the clinically relevant concentration of NA-1. This suggests that the TAT part of the peptides NA-1 and ALVX-144 may in some cases interact with an alternative site of PSD-95, thus not exclusively PDZ1-2. One explanation for the difference between the two peptides may be that it is not possible for the TAT sequence in AVLX-144 to reach the potential secondary site while bound to PDZ1-2, wherein the high affinity and avidity effect could provide less “interaction flexibility” than NA-1, which due to its lower affinity and lack of the PDZ1-2 avidity effect, could switch between lower affinity binding sites, however, more investigations is needed to verify this hypothesis. The ability of known pharmacologically relevant peptides and the cell-penetrating peptide TAT to induce LLPS uncovered here could point to a novel molecular mechanism for some of the neuroprotective effects of these compounds, and in therapeutically relevant concentrations. The difference in binding for NA-1 and AVLX-144 suggests that AVLX-144 is more tightly bound to both PDZ1-2 while NA-1, as expected, favors PDZ2 binding, but is also able to bind PDZ1. The absence of perturbations to the acidic residues in PDZ1, suggests that the residues responsible for TAT-induced LLPS is positioned elsewhere in PSD-95.

Taken together these data suggest that both charge and valency are important for LLPS formation, both for PSD-95 and the ePSD. The presented data also suggest a new angle on the peptide-mediated inhibition of PPIs, implying that peptide-based inhibitors may be designed for and act as LLPS drivers of PPI networks as a novel way of targeting protein–protein interactions.

It will be interesting to see if the ability to induce LLPS in the very simple ePSD system translates into functional effects in the treatment of acute ischemic stroke or similar diseases which rely on the dynamic functions of the PSD.

### Limitations of the study

The limitation of this study relates to the nature of the conducted experiments, which relates to *in vitro* experiments conducted in artificial systems. The study is, therefore, limited to describing the behavior of PSD-95 in combination with the remaining proteins tested in the present study, in the *in vitro* setting of the present study.

## STAR★Methods

### Key resources table

**Table:** 

REAGENT or RESOURCE	SOURCE	IDENTIFIER
**Bacterial and virus strains**		

*E. coli* BL21-DE3-pLysS		

**Chemicals, peptides, and recombinant proteins**		

Ampicillin	HelloBio	HB4322
Chloramphenicol	Sigma	C0378
IPTG	Sigma	10724815001
Trizma® base	Sigma	93362
NaCl	Sigma	S9888
TCEP	Sigma	C4706
cOmplete™ Protease Inhibitor Cocktail	Sigma	11697498001
Deoxyribonuclease I	Sigma	D5025
Imidazole	Sigma	56749
Glutathione Sepharose 4B beads	GE Life Science	17075605
NHS-AlexaFlour647	ThermoFischer	A20006
NHS-AlexaFlour568	ThermoFischer	A20103
C5 Maleimide-AlexaFluor633	ThermoFischer	A20342
AlexaFluor488C5 maleimide	ThermoFischer	A10254
DMSO	Sigma	D2650
YGRKKRRQRRR	TAGCopenhagen	TAT11
biotin-ahx-RMKQLEPKVEELLPKNYHLENE VARLKKLVGGGGSRRTTPV	TAGCopenhagen	mono-Stg
biotin-ahx- RMKQLEDKVEELLSKNYHLENEVARLK KLVGGGGSRRTTPV	TAGCopenhagen	dim-Stg
biotin-ahx-RIKQIEDKIEEILSKIYHIENEIARIK KLIGGGGSRRTTPV	TAGCopenhagen	tri-Stg
YGRKKRRQRRR-NPEG4-di-IETDV	WuXi China	AVLX-144
YGRKKRRQRRR-NPEG4-di-IETAA	In-house	AVLX-144AA
YGRKKRRQRRRIESDV	WuXi China	NA-1
GAITRIPSYRYRYQRR	In-house	Stg_A222-R236_
Folch bovine brain extracts Fractino 1	Sigma	B1502
DiD-C18	Molecular probes	D7757
FL-PSD-95	Zeng et al. (2016a)	PSD-95
PSD-95 61-724	Zeng et al. (2016a)	ΔN-PSD-95
Homer 3 EVH1-CC WT	Zeng et al. (2018)	Homer3
Shank3 NPDZ-HBS-CBS-SAM M1718E	Zeng et al. (2018)	Shank3
GKAP 3GBR-CT	Zeng et al. (2018)	GKAP
SynGAP CC-PBM WT	Zeng et al. (2016a)	SynGAP
Arc 195-364	This paper	GST-Arc

**Recombinant DNA**		

32M3C-PSD-95 FL	Zeng et al. (2016a)	n.a
32M3C-PSD-95 61-724	Zeng et al. (2016a)	n.a
M3C-Homer 3 EVH1-CC WT	Zeng et al. (2018)	n.a
M3C-Shank3 NPDZ-HBS-CBS-SAM M1718E	Zeng et al. (2018)	n.a
32M3C-GKAP 3GBR-CT	Zeng et al. (2018)	n.a
MG3C-SynGAP CC-PBM WT	Zeng et al. (2016a)	n.a
pGEX4T1-Arc 195-364	This paper	n.a

**Software and algorithms**		

Prism 8.3	GraphPad	GraphPad Prism
ASTRA®	Wyatt Technology	n.a
FIDA software 2.0	Fidabio	n.a
ImageJ	https://imagej.nih.gov/ij/index.html	
NMRPipe	Delaglio et al. (1995)	
qMDD	Kazimierczuk and Orekhov (2011)	
CCPNMR analysis	Skinner et al. (2016)	

**Other**		

HisTrap HP 5 mL column	GE Life science	17524701
HiLoad 16/600 Superdex 200 pg	GE Life science	28989335
NAP-5 columns	GE Life science	17085301
Superdex 200 increase 10/300 gl column	GE Lifescience	28990944
FIDA1 instrument	Fidabio	n.a.
Omega POLARstar	BMGlabtech	n.a.
Jasco J1500	Jasco	n.a
Li-COR Odyssey	Li-Cor	n.a
Mini-PROTEAN® TGX™ Precast Protein Gels 10 wells	BioRad	4569036
Mini-PROTEAN® TGX™ Precast Protein Gels 15 wells	BioRad	4569033
Zeiss LSM780 using a 63x NA 1.4 plan apochromat oil objective	Zeiss	n.a.
LabTek	Nunc™, ThermoFischer	155411PK
600 MHz or 750 MHz Bruker Avance III HD spectrometers equipped with QCI or TCI cryo-probes	Bruker	n.a

### Resource availability

#### Lead contact

Further information and requests for resources and reagents should be directed to and will be fulfilled by the lead contact, Kristian Strømgaard (kristian.stromgaard@sund.ku.dk).

#### Materials availability

The pGEX4T1-Rat Arc 195-364 was generated in this study and will be shared upon request. Otherwise, this study did not generate new unique reagents.

### Experimental model and subject details

BL21-DE3-pLysS *E. coli* in LB medium supplemented with 100 μg/mL ampicillin (HelloBio, #HB4322) and 25 μg/mL chloramphenicol (Sigma, C0378). The growth was induced at OD600 0.6-0.8 with 0.5-1 mM IPTG (Sigma 10724815001) and cultures were grown for 16 hrs at 18C shaking at 170 rpm. Cells were harvested at 7000 g and frozen at −80°C until purification.

### Methods details

#### Plasmid preparation

Plasmids encoding FL-PSD-95 (32M3C-PSD-95 FL), ΔN-PSD-95 (32M3C-PSD-95 61-724), Homer3 (M3C-Homer three EVH1-CC WT), Shank3 (M3C-Shank3 NPDZ-HBS-CBS-SAM M1718E), GKAP (32M3C-GKAP 3GBR-CT) and SynGAP (MG3C-SynGAP CC-PBM WT) was a kind gift from Prof. Mingjie. Zhang (The Division of Life Science, Hong Kong University of Science and Technology). In brief, all constructs were previously cloned into a pET32a containing an N-terminal thioredoxin (TRX) tag or a streptococcal protein G (GB1) tag followed by a 6xHis affinity tag and a Prescission C3 protease site, followed by the protein of interest as described in (Zeng et al., 2016a, 2018, 2019).

DNA encoding rat Arc 195-364 (Uniprot: Q63053) was ordered from ThermoFischer with an 3′-*Bam*HI and a *Eco*RI site followed by a FactorXa protease site and the Arc 195-364 coding sequence, followed by a 5′*Hin*dIII, *Not*I and *Xho*I cleavage site. The plasmid was inset into a pGEX4T1 vector using the *Bam*HI and *Xho*I sites, resulting in a construct encoding an N-terminal GST followed by a thrombin and a factorXa protease site followed by rat Arc 195-364.

#### Recombinant protein expression and purification

Plasmids encoding FL-PSD-95 (32M3C-PSD-95 FL 1-724), ΔN-PSD-95 (32M3C-PSD-95 61-724), Homer3 (M3C-Homer three EVH1-CC WT), Shank3 (M3C-Shank3 NPDZ-HBS-CBS-SAM M1718E), GKAP (32M3C-GKAP 3GBR-CT), SynGAP (MG3C-SynGAP CC-PBM WT) and GST-Arc 195-364 were grown in BL21-DE3-pLysS *E. coli* in LB medium supplemented with 100 μg/mL ampicillin (HelloBio, #HB4322) and 25 μg/mL chloramphenicol (Sigma, C0378). The growth was induced at OD_600_ 0.6-0.8 with 0.5-1 mM IPTG (Sigma 10724815001) and cultures were grown for 16 hrs at 18ºC shaking at 170 rpm. Cells were harvested at 7000 g and frozen at -80°C until purification. Pelleted cells were suspended in lysis buffer containing 50 mM Tris (Sigma 93,362) (pH 8.0), 300 mM NaCl (S9888), 1 mM TCEP (Sigma C4706), half a tablet of cOmplete Protease Inhibitor Cocktail (Sigma 11697498001) and 2.5 μg/mL DNase I (Sigma D5025) and sonicated (Branson Sonifier 250, 3 mm round tip, 40% output, 70/30 pulse) on ice until solution became homogeneous. Lysate was centrifuged at 36.000 g for 30 min at 20°C and supernatant was collected and purified using affinity chromatography. 6xHis proteins were purified using a HisTrap HP 5 mL column (GE Life science 17524701) using an imidazole gradient from 10-500 mM imidazole (Sigma 56749). GST-Arc was purified by addition of 700 μL/L culture Glutathione Sepharose 4B beads (GE Life Science, 17075605) in lysis buffer. The GST beads were washed using centrifugation (4000 g for 5 min at 25°C) followed by removal of supernatant and addition of 50 mM Tris (pH 7.4), 300 mM NaCl, 10 mM EDTA, 1 mM TCEP, this step was repeated twice, followed by transfer of beads to a single use gravity column (BioRad 7326008), followed by three on column washes. The protein was eluted using 10 mM reduced GSH (Sigma G4251) in 50 mM Tris (pH 7.4), 300 mM NaCl, 10 mM EDTA, 1mM TCEP. In case of both 6xHis and GST tagged protein, the affinity chromatography was followed by buffer change and purification using size exclusion chromatography (HiLoad 16/600 Superdex 200 pg, GE Life science 28989335) in 50 mM Tris (pH 8.0), 300 mM NaCl, 10 mM EDTA, 1 mM TCEP. Mass and purity was validated using LC-MS and UPLC to be >93% for all purified proteins where concentrated to a suitable concentration, aliquoted and flash frozen in liquid nitrogen. Before fluorescence labeling and use in assays protein was exchanged into PBS-TECP (PBS-TCEP) using NAP-5 columns (GE Life science #17085301), pre-equilibrated in PBS-TCEP.

#### Protein labeling

Before labeling 1 mg of solid dye (NHS-AlexaFlour647, ThermoFischer A20006; NHS-AlexaFlour568, ThermoFischer A20103; C5 Maleimide-AlexaFluor633, ThermoFischer A20342; Alexa Fluor 488C5 maleimide) was diluted into 10μL DMSO (Sigma #D2650) and aliquoted (0.02 mg/tube) and DMSO was evaporated using vacuum evaporation, aliquots were stored at −20°C until usage. For protein labeling, dyes, were dissolved in DMSO and purified protein in PBS-TCEP was incubate with respective dye for 1-2 h. For NHS reactions, the reaction was quenched by addition of 100 mM Tris (pH 7.4). Excess dye and Tris was removed using two consecutive NAP5 columns equilibrated with PBS-TCEP. Protein and dye concentration was measured by NanoDrop 3000 (ThermoFischer). For confocal imaging fluorescent protein was diluted to a final ratio of 1/10 with unlabeled protein.

#### Peptide array synthesis

μSPOT peptide arrays (CelluSpots, Intavis AG, Cologne, Germany) were synthesized using a ResPepSL synthesizer (Intavis AG) on acid labile, amino functionalized, cellulose membrane discs (Intavis AG) containing 9-fluorenylmethyloxycarbonyl-β-alanine (Fmoc-β-Ala) linkers (minimum loading 1.0 μmol/cm). Synthesis was initiated by Fmoc deprotection using 20% piperidine in *N*-methylpyrrolidone (NMP) (1 × 2 and 1 × 4 μL, 3 and 5 min, respectively) followed by washing with dimethylformamide (DMF, 7 × 100 μL per disc) and ethanol (EtOH, 3 × 300 μL per disc). Peptide chain elongation was achieved using 1.2 μL of coupling solution consisting of preactivated amino acids (0.5 M) with 2-(1-benzotriazole-1-yl)-1,1,3,3-tetramethyluronium hexafluorophosphate (0.5 M) and *N*,*N*-diisopropylethylamine (DIPEA) in NMP (2:1:1, amino acid:HBTU:DIPEA). The couplings were carried out 7 times (20 min for the first coupling and 30 min for the rest), and subsequently, the membrane was capped twice with capping mixture (5% acidic anhydride in NMP), followed by washes with DMF (7 × 100 μL per disc). After chain elongation, final Fmoc deprotection was performed with 20% piperidine in NMP (3 × 4 μL, 5 min each), followed by six washes with DMF, subsequent N-terminal acetylation with capping mixture (3 × 4 μL, 5 min each) and final washes with DMF (7 × 100 μL per disc) and EtOH (7 × 200 μL per disc). Dried cellulose membrane discs were transferred to 96 deep-well blocks and were treated with the side-chain deprotection solution consisting of 80% trifluoracetic acid (TFA), 12% DCM, 5% H_2_O, and 3% triisopropylsilane (TIPS) (150 μL per well) for 1.5 h at room temperature. Afterwards, the deprotection solution was removed, and the discs were solubilized overnight at room temperature using a solvation mixture containing 88.5% TFA, 4% trifluoromethansufonic acid (TFMSA), 5% H_2_O, and 2.5% TIPS (250 μL per well). The resulting peptide-cellulose conjugates were precipitated with ice-cold diethyl ether (1 mL per well) and spun down at 1000 rpm for 90 min, followed by an additional wash of the formed pellet with ice-cold diethyl ether. The resulting pellets were re-dissolved in dimethyl sulfoxide (DMSO, 500 μL per well) to give final stocks, which were transferred to a 384-well plate and printed (in duplicates) on white coated CelluSpots blank slides (76 × 26 mm, Intavis AG) using a SlideSpotter robot (Intavis AG).

#### celluSPOT array

Prior to incubation with the protein or proteins of interest, the array was washed 3 times in PBS, and the surface was blocked with BSA (5 mg/mL) in PBS for 1 hour at room temperature under gentle oscillation, whereafter the array was washed 3 times in PBS, and the indicated amount of protein was added in PBS + TCEP (1 mM), and was incubated under gentle oscillation. Before imaging the membranes were washed 3 times in PBS.

Images were obtained using a Li-COR Odyssey scanner using the 700 nm fluorescence imaging setting. Images were exported and individual spot intensities were analyzed using ImageJ. Normalized intensities were obtained by normalization of the intensity of each spot to the maximal and minimal value obtained in each array. The normalized values were pooled and the average value is reported with the SD or SEM where appropriate. Relative intensities were obtained for GKAP, Shank3, Homer3 and SynGAP, where the raw intensities were corrected for differences in degree of labeling for each protein to obtain the relative intensity of each spot. Raw intensities are given as the intensities obtained from the image quantification.

#### Peptide synthesis

Purified (>95% purity) TAT11 (YGRKKRRQRRR), mono-Stg (biotin-ahx-RMKQLEPKVEELLPKNYHLENEVARLKKLVGGGGSRRTTPV), dim-Stg (biotin-ahx- RMKQLEDKVEELLSKNYHLENEVARLKKLVGGGGSRRTTPV), tri-Stg (biotin-ahx-RIKQIEDKIEEILSKIYHIENEIARIKKLIGGGGSRRTTPV) were ordered and from TAGCopenhagen (Denmark). Purified (>95% purity) AVLX-144 was ordered from WuXi peptides (China). UCCB-125 and AVLX-144-AA were synthesized in house using previously reported synthesis (Bach et al., 2009, 2012).

The synthesis of the Stg_A222-R236_ peptide (GAITRIPSYRYRYQRR), using Fmoc-based solid phase peptide synthesis, was carried on a Prelude X, induction heating assisted, peptide synthesizer (Gyros Protein Technologies, Tucson, AZ, USA) with 10 mL glass reaction vessel using preloaded Wang-resins (100–200 mesh). All reagents were prepared as solutions in DMF: Fmoc-protected amino acids (0.2 M), *O*-(1*H*-6-chlorobenzotriazole-1-yl)-1,1,3,3-tetramethyluronium hexafluorophosphate (HCTU, 0.5 M) and DIPEA, 1.0 M. Coupling steps were carried out using the following protocol: deprotection (20% piperidine in DMF, 2 × 2 min, room temperature, 300 rpm shaking), coupling (2 × 5 min, 75°C, 300 rpm shaking, for Arg and His couplings 2 × 5 min, 50°C, 300 rpm shaking). Amino acids were double coupled using amino acid/HCTU/DIPEA (ratio 1:1.25:2.5) in 5-fold excess over the resin loading to achieve peptide sequence elongation.

N-terminal labeling of peptide A222-R236 with 5 (and 6)-carboxytetramethylrhodamine (TAMRA, Anaspec Inc.) was performed on resin, by coupling TAMRA for 16 h at room temperature using a mixture of 1.5:1.5:3 [TAMRA:benzotriazol-1-yloxy)tripyrrolidinophosphonium hexafluorophosphate (PyBOP): DIPEA] in NMP (Witte et al., 2013). The coupling was finalized with extensive washes of resin with DMF and DCM.

The synthesized peptides were cleaved from the resin using a mixture of 90:2.5:2.5:2.5:2.5 (TFA:H_2_O:TIPS:1,2-ethanedithiol (EDT):thioanisole) for 2 h at room temperature. After cleavage the peptide was precipitated with an ice-cold diethyl ether and centrifuged at 3500 rpm for 10 min at 4°C. The resulting peptide precipitate was re-dissolved in 50:50:0.1 (H_2_O:CH_3_CN:TFA) and lyophilized. Purification of the crude peptide was performed with a preparative reverse phase high-performance liquid chromatography (RP-HPLC) system (Waters) equipped with a reverse phase C18 column (Zorbax, 300 SB-C18, 21.2 × 250 mm) and using a linear gradient with a binary buffer system of H_2_O:CH_3_CN:TFA (A: 95:5:0.1; B: 5:95:0.1) (flow rate 20 mL/min). The collected fractions were characterized by LC-MS. The purity (≥95%) of the fractions was determined at 214 nm on RPUPLC. The final lyophilized products were used in further experiments.

#### Liposome preparation

For preparation of liposomes we used Folch bovine brain extracts (Fraction 1, Sigma B1502), and 1,1′-dioctadecyl-3,3,3′,3′-tetramethyl-indodicarbocyanine perchlorate (DiD) (Molecular probes, D7757). The liposomes were prepared using a previously described lipid hydration method (Hatzakis et al., 2009). In brief, lipids (Bovine Folch fraction, Sigma) dissolved in chloroform were thoroughly mixed in a glass vial, at a molar ratio of 99.5:0.5 (Brain:DiD). The solution was dried under nitrogen flow and incubated in vacuum overnight. Liposomes were rehydrated by carefully adding a 200 mM D-Sorbitol solution to the lipid film, for a final lipid concentration of 1 g/L. The mixture was re-suspended at 37 °C, before the liposomes were subjected to ten freeze-thaw cycles to minimize multi-lamellarity by immersion in liquid nitrogen followed by thawing in a water bath. After freeze-thaw cycles, liposomes were extruded seven times through a single Isopore polycarbonate membrane with a pore size of 1000 nm from Millipore in an Avanti Mini-extruder. The liposomes were flash-frozen in liquid nitrogen and stored at −21 °C. We have previously shown using electron microscopy imaging that the mult-ilamellarity of our liposome preparations is negligible (<5%) (Hatzakis et al., 2009). Liposomes were diluted in PBS to 0.002 mg/mL and were added to the array and incubated for 1 hour, whereafter the membrane was washed three times in PBS, and imaged using a Li-COR Odyssey gel scanner using the 700 nm fluorescence imaging setting. Images were exported and individual spot intensities were analyzed using ImageJ as described above. For each array, related to the liposome binding, the intensities were normalized to 100% for the 228-242 peptide and 0% for the average background value, measured outside the spots.

#### Size exclusion chromatography

Before analytical SEC, stocks in PBS-TECP were mixed to desired concentrations in PBS-TECP and incubated for 20 min at room temperature before being run on a Superdex 200 increase 10/300 gl column (GE Lifescience 28990944) monitoring the Absorbance at 220 nm, 260 nm and 280 nm. The resulting absorbance trace at 260 (peptides alone) 280 nm (in complex with PSD-95) was plotted and normalized to the maximal absorbance for each condition. For peptide and protein containing samples, the data was normalized to the maximal absorbance of the protein sample in absence of peptide. Data was plotted using GraphPad Prism 8.3.

#### SEC multi angle light scattering (MALS)

SEC-MALS was done using an Agilent HPLC equipped with a Wyatt MALS setup, where 50 μL of 50 μM PSD-95 incubated with 150 μM of indicated peptide was loaded onto a Superdex200 Increase 10/300 column equilibrated in 50 mM Tris (pH 7.5), 200 mM NaCl, 1 mM TCEP and both absorbance, refractive index and light scattering data was collected. Resulting data was analyzed and molecular weight was calculated using the ASTRA software package, data was plotted using GraphPad Prism 8.3.

#### Flow induced dispersion analysis (FIDA)

FIDA was carried out using intrinsic fluorescence, using the standard protocol recommended by the manufacturer, in short, PSD-95 (12 μM) in absence or presence of 12 μM or 36 μM peptide was loaded to the FIDA1 instrument, the protein containing solution was used as injectant and the protein with peptide was used as analyte solution. The diffusion of the complex could then be observed using intrinsic fluorescence, and the hydrodynamic radius was calculated using the FIDA software 2.0 using a single Gaussian distribution fit, at 75% and curve smoothing. Resulting hydrodynamic radius was plotted using GraphPad Prism 8.3.

#### Fluorescence polarization

Fluorescence Polarization (FP) saturation binding (also described in (Bach et al., 2012; Madsen et al., 2005)) was carried out in a buffer containing 50 mM Tris (pH 8.0), 300 mM NaCl, 10 mM EDTA, 1 mM TCEP, using an increasing amount of protein incubated with a fixed concentration of fluorescently labeled peptides as indicated. Competition FP was done at a fixed concentration of PSD-95 and a bivalent fluorescent tracer, AB-143 (Bach et al., 2012), against an increasing concentration of unlabeled peptide. After mixing the 96-well plate (a black half-area Corning Black non-binding) was incubated 20 min on ice after which the fluorescence polarization was measured directly on a Omega POLARstar plate reader using excitation filter at 488 nm and long pass emission filter at 535 nm. The data was plotted using GraphPad Prism 8.3 and fitted to the either a single exponential binding curve or a sigmoidal single site binding model for saturation experiments or One site competition for competition experiments. K_i_’|'s were automatically calculated using the Cheng-Prusoff equation. All binding isotherms were repeated at least three technical replicates or as indicated in figure legend.

#### Circular dichroism (CD)

Before CD measurements samples were diluted into 50 mM NaPi buffer (pH 8.0) to a suitable concentration, Stg peptides 8 μM was used and for NA-1 and AVLX-144 10 μM was used. CD measurements were done using a Jasco J1500 at 25°C with a quartz cell with a 1 mm path length quartz cuvette. Each spectrum was recorded from 260-190 nm at a 0.1 nm step resolution and a scan speed of 50-100 nm/min, each presented spectrum is the average of three scans. The resulting mDEG signal was converted into molar ellipticity, *θ* (deg ⋅ cm^2^ ⋅ dmol) using the equation *θ* = (mDEG∙10^6^)/(C∙N∙L), where mDEG is the measured signal, C is the protein concentration in μM, N is the number of residues in the protein, L is the cuvette path length in mm. The resulting CD spectra were plotted using GraphPad Prism 8.3.

#### SDS-PAGE sedimentation assay

Proteins were all mixed in the desired concentration in PBS-TCEP and equilibrated for 10 min before centrifugation at 20,000 g for 15 min at 25°C using a temperature-controlled table top centrifuge. Following centrifugation, the supernatant was collected and the pellet was re-suspended in an equal amount of PBS-TCEP, usually 50 μL. To ensure proper suspension of LLPS samples were vortexed before addition of SDS buffer boiling at 95°C for 5 min. Supernatant and pellet fractions were run on any kD Mini-PROTEAN TGX Precast Protein Gels (10 or 15 wells, BioRad 4569036 or 4569033). Gels were imaged using a Li-COR Odyssey gel scanner and band intensities were analyzed using ImageJ. Significance was evaluated using one-way ANOVA with Dunnett post-test, one-way ANOVA with Tukey post-test or a two-way ANOVA with Dunnett post-test.

#### Confocal microscopy on LLPS droplets

Confocal microscopy was done using a Zeiss LSM780 using a 63x NA 1.4 plan apochromat oil objective using Argon 488 nm 25 mW, 543 nm HeNe 1.2 mW and 633 nm HeNe 5mW lasers using a detection wavelength of 490-538 nm for the 488 channel, 556-627 nm for the 543 channel, 636-758 for the 633 channel. Images were acquired using averaging of four line scans and 12-bit. The LLPS droplets were prepared in the desired concentration in PBS-TCEP at desired pH, mostly pH 7.4 unless stated otherwise, and added to an untreated lab tec (155411PK, Nunc, ThermoFischer) and imaged after being allowed to settle for 15 min at 25°C. For samples containing fluorescent protein or peptide the content of fluorescent protein or peptide was kept at 1-10% of indicated total protein or peptide concentration. Fluorescence after photo bleaching (FRAP) experiments was done by bleaching of the 488nm or 647 nm channel, normalizing the fluorescence intensity to ROI intensity before bleaching to one and immediately after bleaching to 0.

#### NMR spectroscopy

All NMR spectra were recorded on 600 MHz or 750 MHz Bruker Avance III HD spectrometers equipped with QCI or TCI cryo-probes at 25°C in 50 mM Tris pH 8, 200 mM NaCl, 1 mM TCEP, 10% D2O and 250 μM DSS. Spectra were processed with NMRPipe (Delaglio et al., 1995) or qMDD (Kazimierczuk and Orekhov, 2011) if non-uniform sampling was used for the acquisition and analyzed using CCPNMR analysis (Skinner et al., 2016). Amide nitrogen and proton chemical shift assignments were kindly shared by Prof. Mingjie Zhang and validated using HNCA and HN(CO)CA experiments on a sample with 300 μM ^13^C^15^N PSD-95 PDZ1-2. Ligand titrations were followed by ^1^H-^15^N-HSQC experiments recorded on 100 μM ^15^N PSD-95 and ligand concentrations ranging from 500 μM to 4 μM. Combined chemical shift perturbations were calculated between the unbound and the bound states using.

NMR titrations at pH 7.4, 6.4 and 5.4 were followed by 1H-15N-HSQC experiments using 40 μM PDZ1-2 in 50 mM Tris pH (7.4, 6.4 or 5.4), 200 mM NaCl, 1 mM TCEP, 10% D2O and 250 μM DSS. Between each spectrum the pH was changed by titration with 1 M HCl.

### Quantification and statistical analysis

In all cases images were imported and quantified using ImageJ. For confocal and celluSPOT images raw intensities were measure in region of interest (ROIs), and data was exported into GraphPad Prism and analyzed.

Statistics was done using one-way or two-way ANOVA with Dunnett post-test. ∗, p<0.05; ∗∗, p< 0.01; ∗∗∗, p< 0.001; ∗∗∗∗ p< 0.0001.

## Data Availability

Any additional information required to reanalyse the data, including data and code reported in this paper is available from the lead contact upon request.
