# Supplementary material for: Bidirectional protein–protein interactions control liquid–liquid phase separation of PSD-95 and its interaction partners
Source: iScience. 2022 Jan 25;25(2):103808. doi: 10.1016/j.isci.2022.103808 (PMC8844826; doi:10.1016/j.isci.2022.103808)
Supplement: Document S1. Figures S1–S12 [file mmc1.pdf]

iScience, Volume *25*

Supplemental information

Bidirectional protein–protein interactions control liquid–liquid phase separation of PSD-95 and its interaction partners

Nikolaj Riis Christensen, Christian Parsbæk Pedersen, Vita Sereikaite, Jannik Nedergaard Pedersen, Maria Vistrup-Parry, Andreas Toft Sørensen, Daniel Otzen, Kaare Teilum, Kenneth Lindegaard Madsen, and Kristian Strømgaard

SUPPLEMENTAL INFORMATION TITLES AND LEGENDS:

SUPPLEMENTARY FIGURES AND TABLES:
Supplementary figures

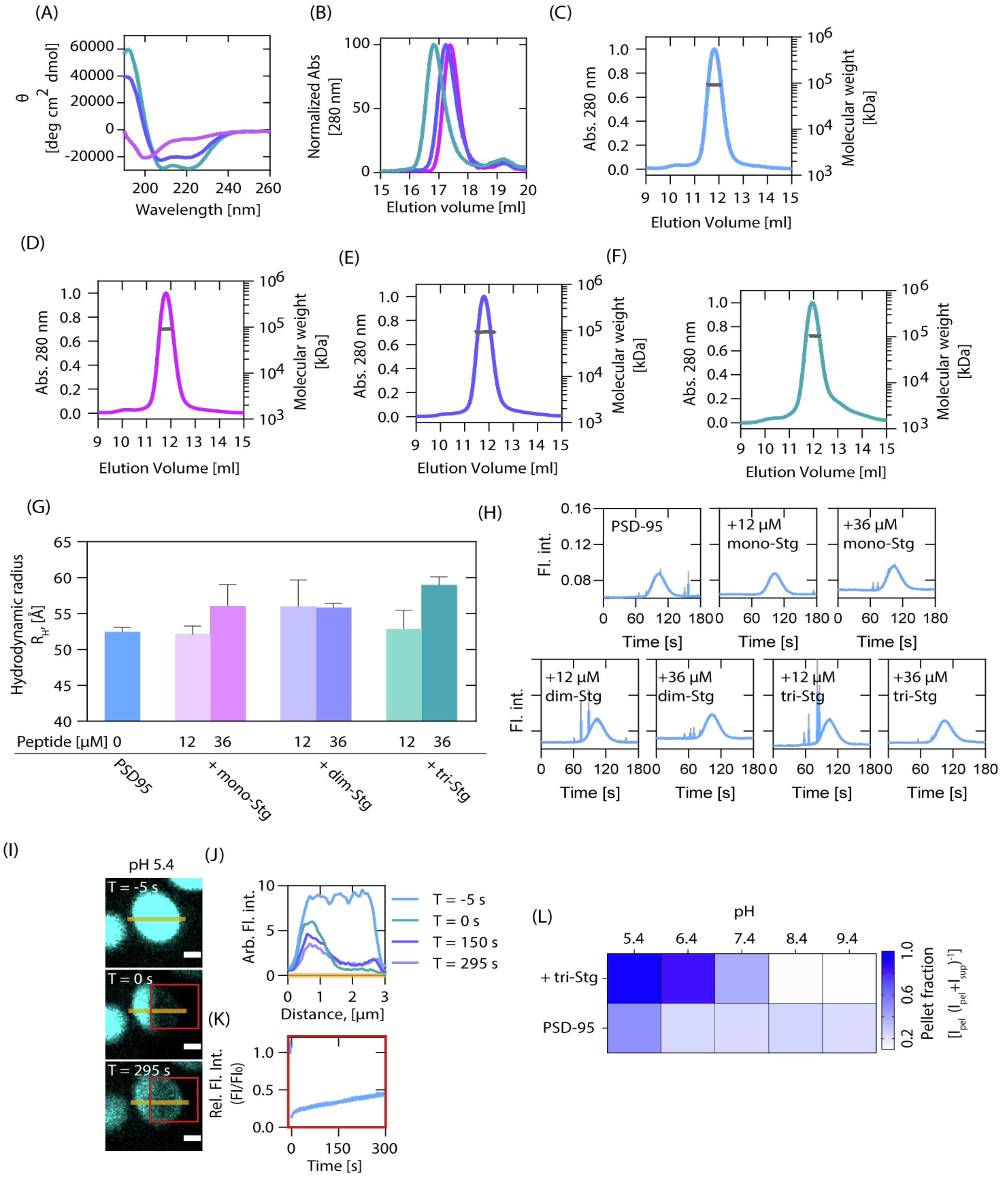

Figure S1. Secondary structure and size distributions of mono-Stg, dim-Stg and tri-Stg in absence and presence of PSD-95, related to figure 1, (A) Circular dichroism of mono-Stg (purple), dim-Stg (blur) and tri-Stg (green) shows helical structure for dim-Stg and tri-Stg, while mono-Stg adopts a random coil like structure. (B) Size exclusion chromatography of mono-Stg (purple), dim-Stg (blue) and tri-Stg (green) indicates an increase in hydrodynamic radius for tri-Stg over mono-Stg and dim-Stg.(C-F) SEC-MALS elution profiles of 50 μM TRX-PSD-95 in absence (C) or presence of 150 μM mono-Stg (D), dim-Stg (E) or tri-Stg (F) and fitted molecular weights. (G) FIDA obtained hydrodynamic radius of 12 μM TRX-PSD-95 (blue) in complex with mono-Stg (purple), dim-Stg (blue) or tri-Stg (green) indicates a slight increase in size for the complex, as also indicated from SEC-MALS data (C-F), errorbars are shown as SEM of N=3. (H) Average FIDA taylorgrams of (J), show spikes in signal possibly due to LLPS droplets in some mixtures. Error is shown in grey intervals as SEM of N=3. (I) Representative FRAP imagesof LLPS droplet PSD-95 at pH 5.4. Scale bars indicate 1 um. (J) Line intensity profile of PSD-95 at indicated timepoints, which show a time dependent reduction in PSD-95 and minor recovery of PSD-95 signal. (K) Quantification of PSD-95 droplets FRAP recovery at pH 5.4 suggests some dynamics in the PSD-95 droplets. (L) Heat-map representation of SDS-PAGE sedimentation assay with full length PSD-95 in absence or presence of tri-Stg indicates strong pH dependency of multivalent LLPS formation.

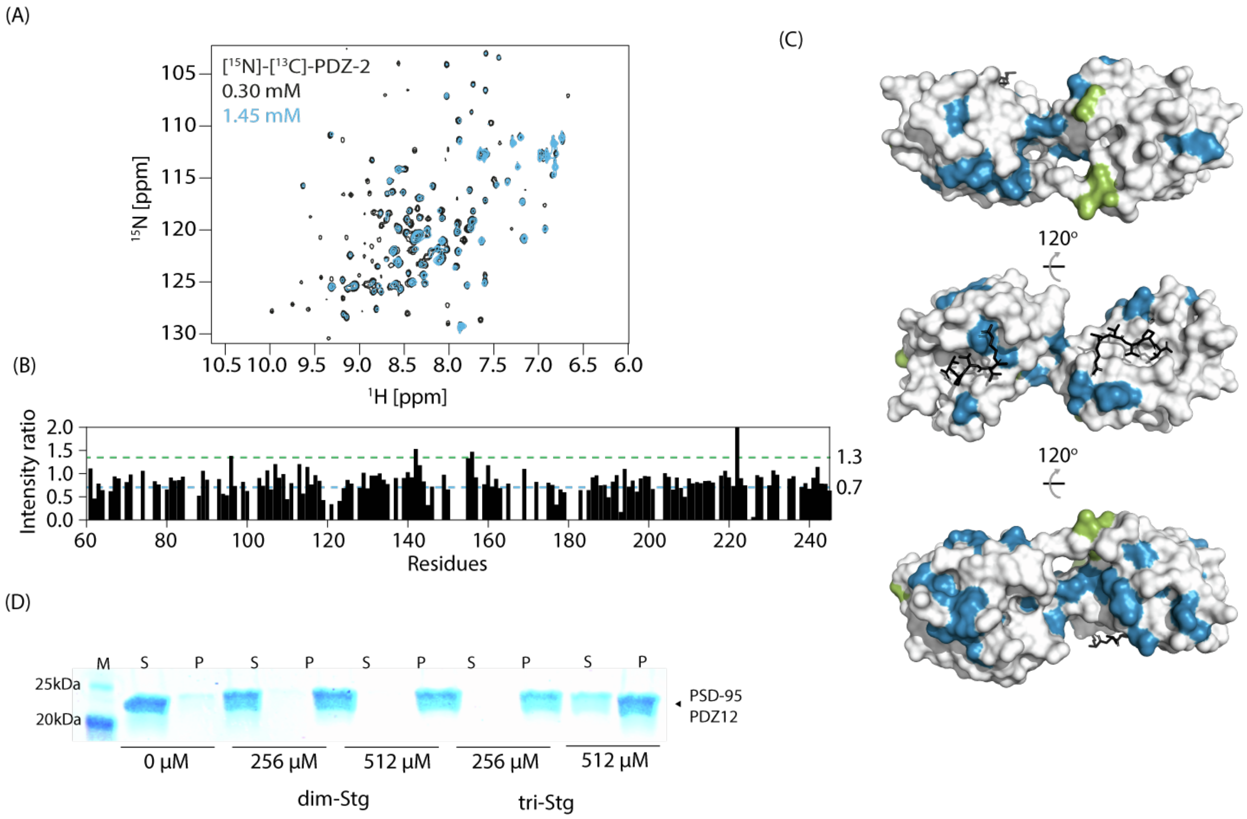

Figure S2. PDZ-12 HSQC intensity changes at mM concentrations, related to figure 2 (A) ^{1}H-^{15}N-HSQC spectra overlay of ^{15}N-labelled PSD-95 PDZ12 at 1.45 mM (teal) and 300 µM (black). (B) Peak intensity ratios of ^{1}H-^{15}N-HSQC spectra in (B), where peaks were normalized according to concentrations and the ratio was taken as $I_{peak,1.45\ mM}$ / $I_{peak,0.3\ mM}$. (C) Residues with intensity ratio change over 30%, was mapped onto the structure of PDZ1-2 (PDB: 3GSL), with intensity decrease over 30% (<0.7 in (B)) are presented in teal and intensity increases over 30% (>1.3 in (B)) are presented in green and black ligands represents RTTPV which was docked into the PDZ binding pocket of both PDZ1 and PDZ2 using alignment to PDB ID 3JXT. (D) SDS-PAGE sedimentation assay of PDZ1-2 in absence or presence of indicated peptide.

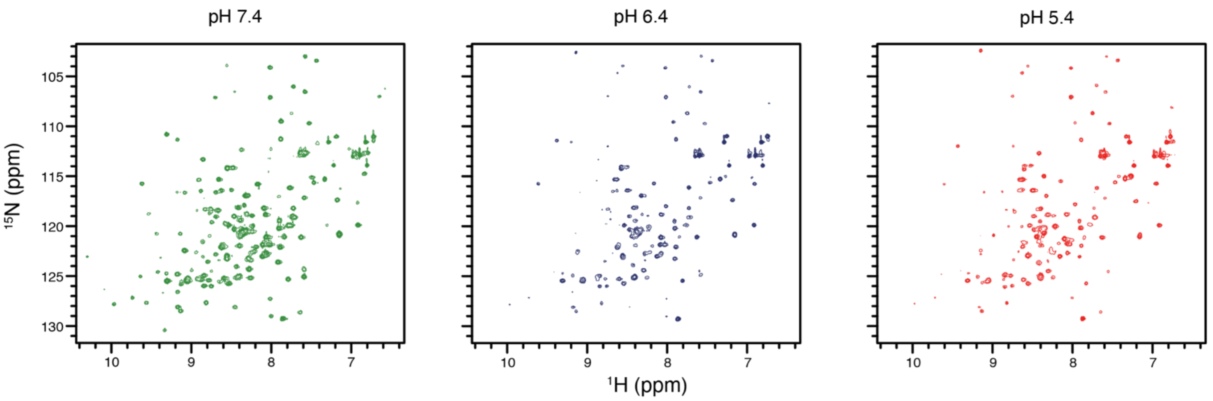

Figure S3. **PDZ-12 HSQC intensity changes at varying pH, related to figure 1 and 2** 1H-15N-HSQC spectra of PDZ1-2 at pH 7.4 (green), pH 6.4 (purple) and pH 5.4 (red) in 50 mM Tris, 200 mM NaCl, 1 mM TCEP, 10 % D2O and 250 μM DSS at 298 K.

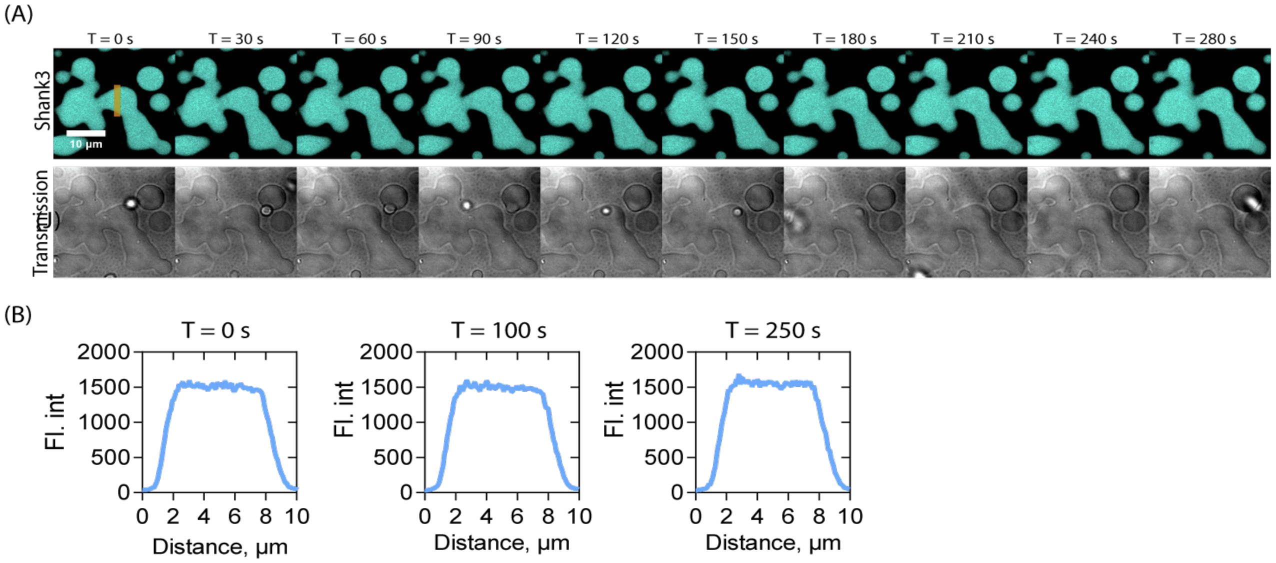

Figure S4 H-S-G-S fluorescence intensity changes over time upon addition of PBS, related to figure 3
(A) Time series of H-S-G-S condensate after addition of PBS. (B) Line profile of Shank3 at indicated time points shows no difference in Shank3 intensity upon addition of PBS. Orange line in (A) indicates line segment.

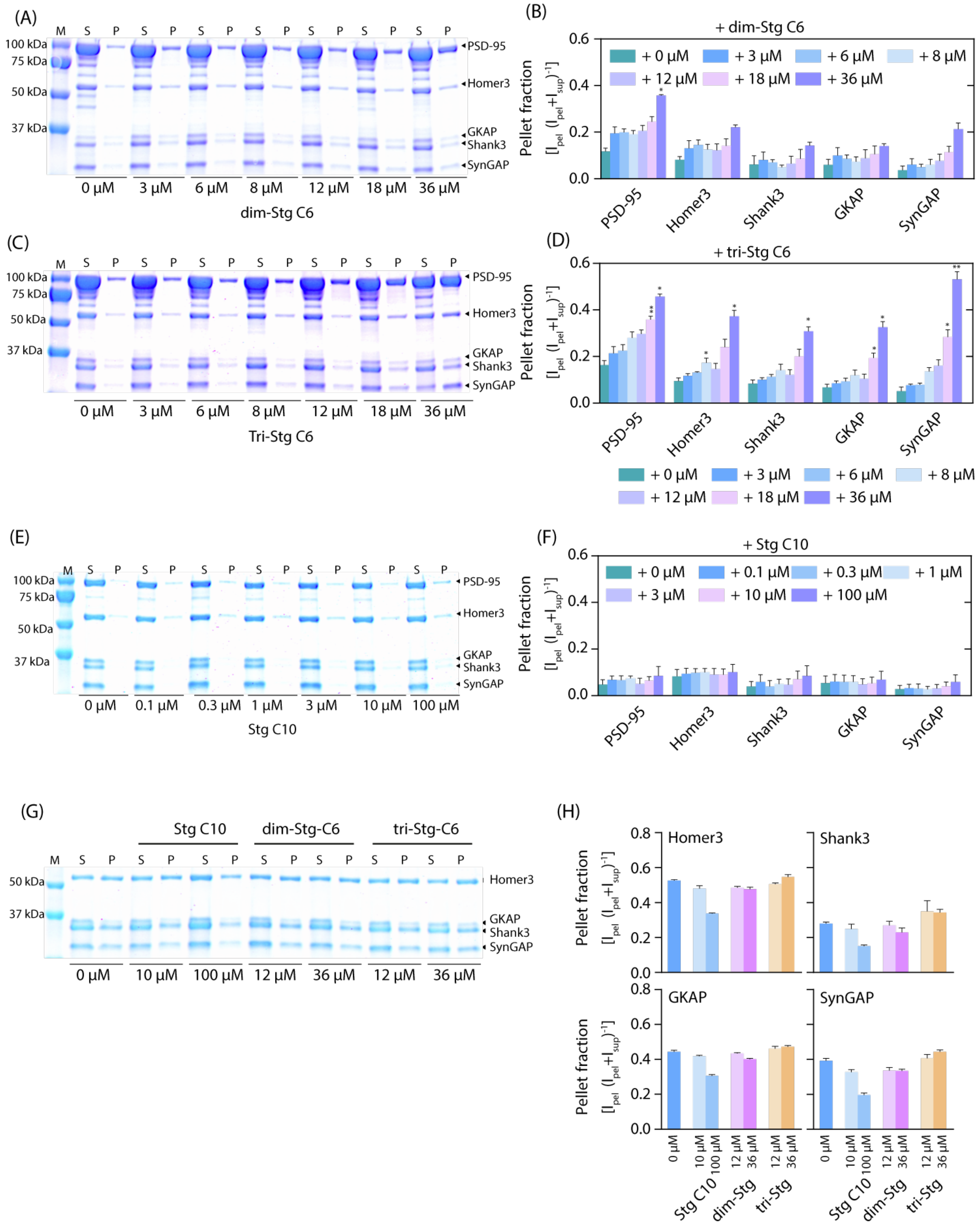
(A)
M S P S P S P S P S P S P S P
100 kDa
75 kDa
50 kDa
37 kDa
PSD-95
Homer3
GKAP
Shank3
SynGAP
0 μM 3 μM 6 μM 8 μM 12 μM 18 μM 36 μM
dim-Stg C6
(B)
+ dim-Stg C6
+ 0 μM + 3 μM + 6 μM + 8 μM + 12 μM + 18 μM + 36 μM
Pellet fraction
$[I_{pel} (I_{pel}+I_{sup})^{-1}]$
0.6 0.4 0.2 0.0
PSD-95 Homer3 Shank3 GKAP SynGAP
(C)
M S P S P S P S P S P S P S P
100 kDa
75 kDa
50 kDa
37 kDa
PSD-95
Homer3
GKAP
Shank3
SynGAP
0 μM 3 μM 6 μM 8 μM 12 μM 18 μM 36 μM
Tri-Stg C6
(D)
+ tri-Stg C6
Pellet fraction
$[I_{pel} (I_{pel}+I_{sup})^{-1}]$
0.6 0.4 0.2 0.0
PSD-95 Homer3 Shank3 GKAP SynGAP
+ 0 μM + 3 μM + 6 μM + 8 μM + 12 μM + 18 μM + 36 μM
(E)
M S P S P S P S P S P S P S P
100 kDa
75 kDa
50 kDa
37 kDa
PSD-95
Homer3
GKAP
Shank3
SynGAP
0 μM 0.1 μM 0.3 μM 1 μM 3 μM 10 μM 100 μM
Stg C10
(F)
+ Stg C10
+ 0 μM + 0.1 μM + 0.3 μM + 1 μM + 3 μM + 10 μM + 100 μM
Pellet fraction
$[I_{pel} (I_{pel}+I_{sup})^{-1}]$
0.6 0.4 0.2 0.0
PSD-95 Homer3 Shank3 GKAP SynGAP
(G)
Stg C10 dim-Stg-C6 tri-Stg-C6
M S P S P S P S P S P S P S P
50 kDa
37 kDa
Homer3
GKAP
Shank3
SynGAP
0 μM 10 μM 100 μM 12 μM 36 μM 12 μM 36 μM
(H)
Homer3
Shank3
GKAP
SynGAP
Pellet fraction
$[I_{pel} (I_{pel}+I_{sup})^{-1}]$
0.8 0.6 0.4 0.2 0.0
0 μM 10 μM 100 μM 12 μM 36 μM 12 μM 36 μM
Stg C10 dim-Stg tri-Stg

Figure S5 SDS-PAGE sedimentation and quantification of ePSD and H-S-G-S, related to figure 4 (A-D) SDS-PAGE sedimentation and quantification of 5xePSD (3 μM H-S-G-S, 10 μM PSD-95) incubated with increasing amounts of dim-Stg (A-B) or tri-Stg (C-D). (E-F) SDS-PAGE sedimentation and quantification of 5xePSD (3 μM H-S-G-S, 3 μM PSD-95) incubated with increasing amounts of StgC10. Heatmap representation of A-D is shown in Figure 3H-I. (G) SDS-PAGE sedimentation assay with 3 μM H-S-G-S condensate incubated with indicated amount of Stg derived peptides. (H) Quantification of (G) shows no effects of peptide addition on condensate formation. Error bars are shown as SEM of n=3.

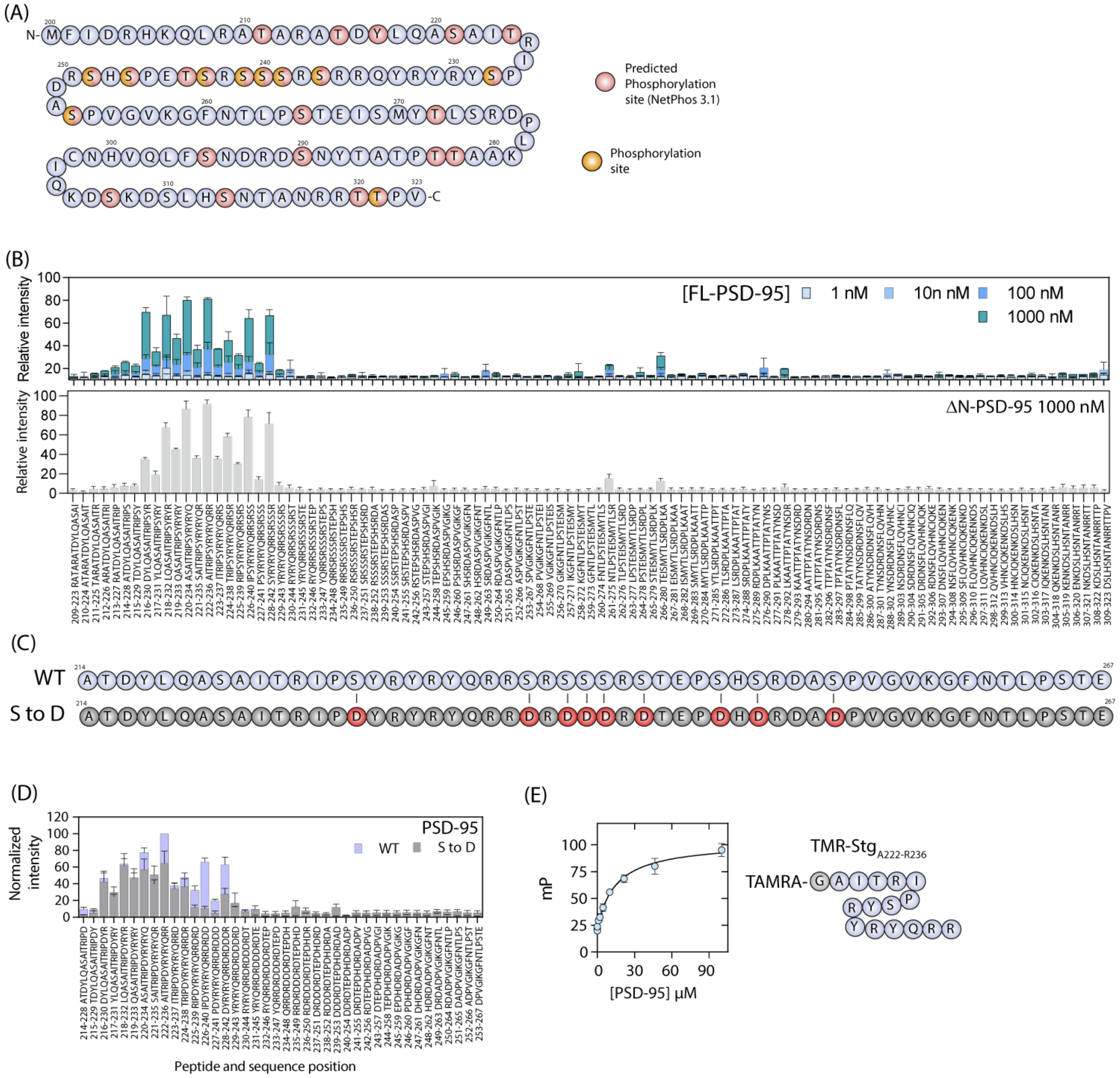

Figure S6 Quantification of arrays of Stg C-terminal peptides, related to figure 5 (A) Primary sequence of Stg cytoplasmic C-terminal (200-323) with known and predicted phosphorylation sites indicated. (B) Quantification of arrays of Stg C-terminal peptides (16-mers) when incubated with indicated protein. Primary sequence of peptides is indicated below each bar. Error bars are shown as SD of duplicate measurements. FL-PSD-95 and ΔN-PSD-95 was labelled with Alexa633. (C) Primary sequence with indicated Ser to Asp (S-to-D) mutations (red) mimicking the previously reported Ser phosphorylation. (D) Comparison between PSD-95 binding to WT (blue) and S-to-D (grey) peptide array. (E) FP saturation binding of TAMRA labelled $Stg_{A222-R236}$ to FL-PSD-95. Error bars are shown as SEM of n=3.

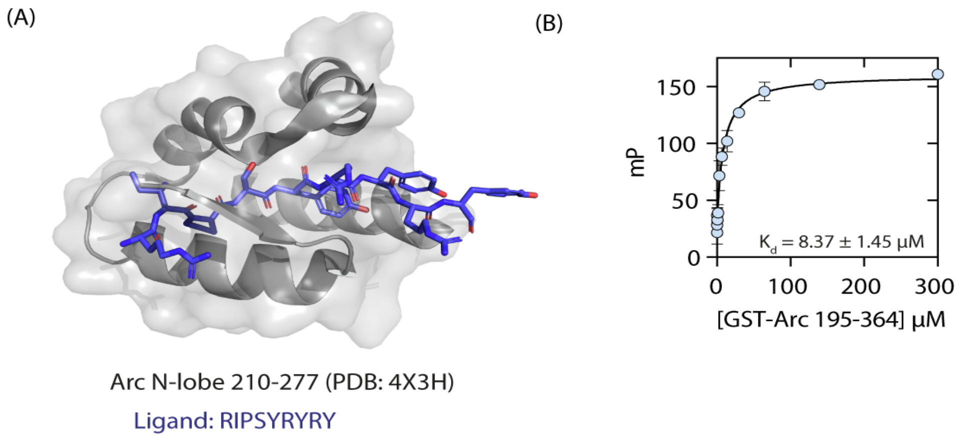

Figure S7 Fluorescence polarization binding of Arc N-/C-lobe to TAMRA labelled $Stg_{A222-R236}$, related to figure 5 (A) X-ray crystal structure (PDB: 4X3H) of Arc N-lobe (grey) binding to Stg peptide RIPSYRYRY (blue), (Zhang et al., 2015). (B) Fluorescence polarization binding of GST fused rat Arc N-/C-lobe (Residues 195-364, Uniprot: Q63053). Fitting was done using GraphPad Prism 8.3, using a single binding site model. Error bars are shown as SEM of n=3.

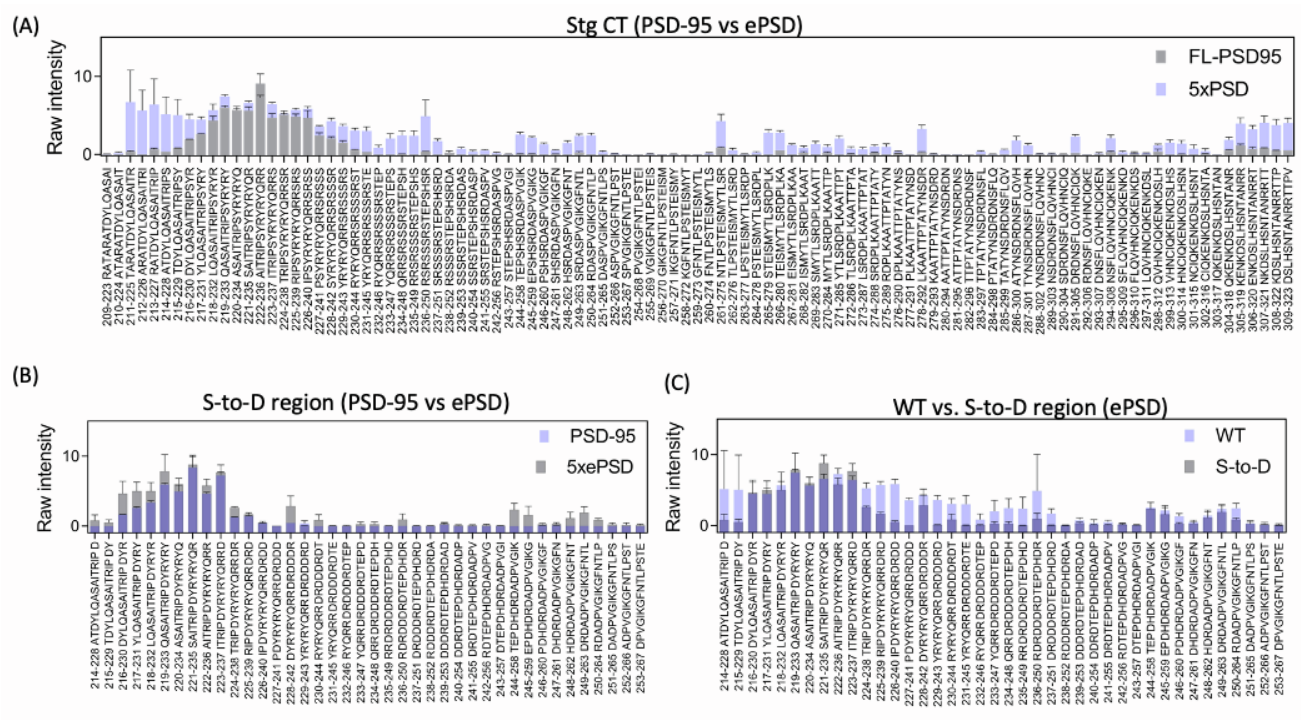

Figure S8 Quantification of arrays of Stg C-terminal peptides, raw intensity, related to figure 5 (A) Raw intensity values of arrays of Stg C-terminal peptides (16-mers) when incubated with indicated PSD-95 or ePSD. Primary sequence of peptides is indicated below each bar. Error bars are shown as SD of duplicate measurements (PSD-95) or six replicates (ePSD). (B) Comparison between raw intensity values for PSD-95 (blue) and ePSD (grey) binding to the S-to-D region. Error bars are shown as SD of duplicate measurements (PSD-95) or six replicates (ePSD). (C) Comparison between raw intensity values for ePSD binding to the WT (blue) or S-to-D (grey) peptides. Errorbars show SEM of n=6.

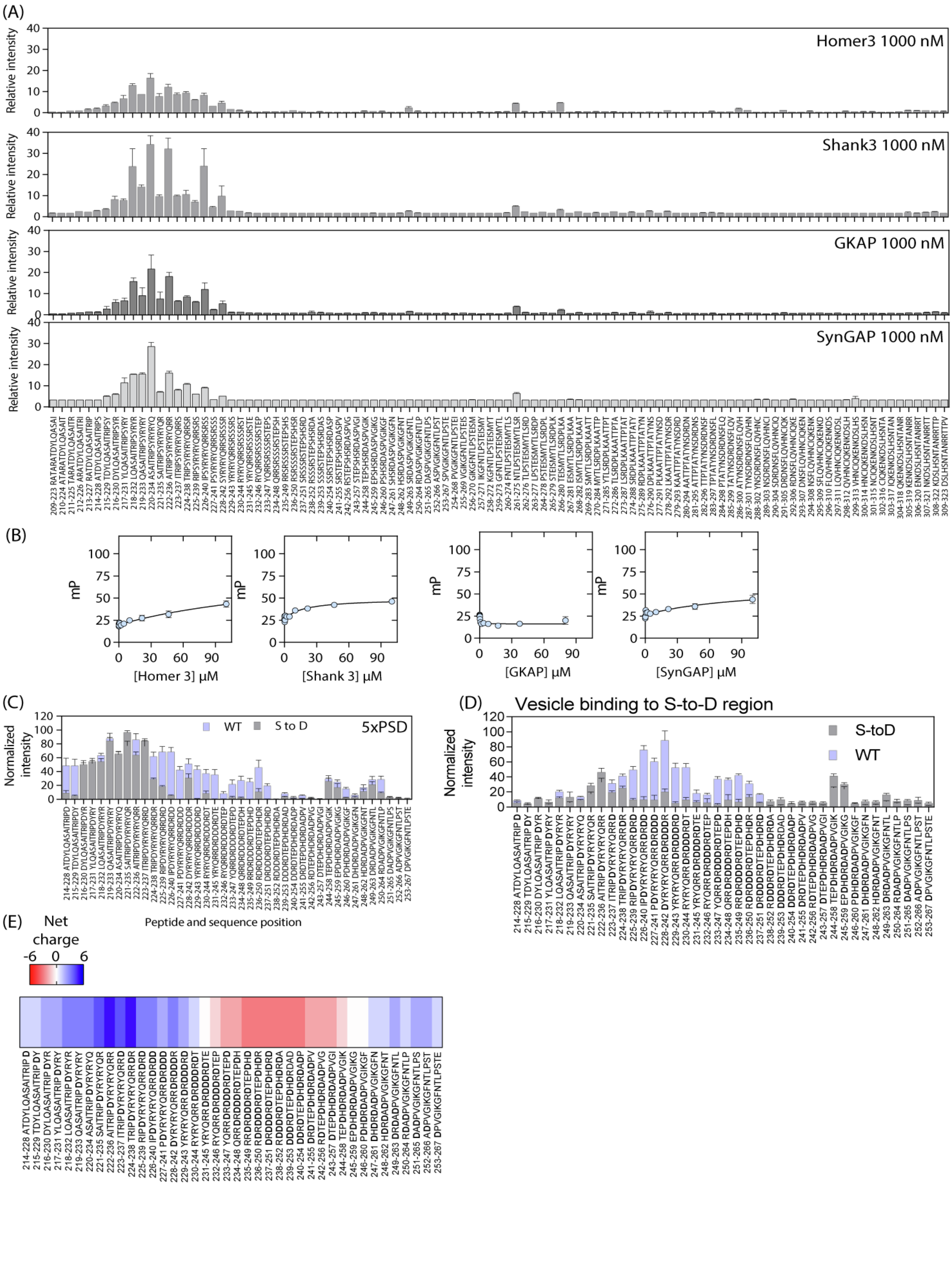

(A)
Homer3 1000 nM
Shank3 1000 nM
GKAP 1000 nM
SynGAP 1000 nM
Relative intensity
(B)
mP
[Homer 3] μM
[Shank 3] μM
[GKAP] μM
[SynGAP] μM
(C)
Normalized intensity
WT
S to D
5xPSD
Peptide and sequence position
(D)
Vesicle binding to S-to-D region
Normalized intensity
S-toD
WT
(E)
Net charge
-6
0
6

Figure S9 Quantification of arrays of Stg C-terminal peptides for Homer3, Shank3, GKAP and SynGAP, related to figure 5 (A) Quantification of arrays of Stg C-terminal peptides (16-mers) when incubated with indicated protein. Primary sequence of peptides is indicated below each bar. Error bars are shown as SD of duplicate measurements. Homer3, Shank3, GKAP and SynGAP were labelled with NHS-Alexa647. (B) Fluorescence polarization binding of TMR-$Stg_{A222\text{-}R236}$ to Homer3, Shank3, GKAP or SynGAP. Fitting was done using GraphPad Prism 8.3, using a single binding site model. Error bars are shown as SEM of n=3. (C) Comparison between ePSD binding to WT (blue) and S-to-D (grey) peptide array. (D) Binding to the S-to-D region for vesicles. Signal was normalized to maximal signal of WT peptides. Error bars are shown as SEM of n=6. WT data in is a regional crop from data shown in Figure 4C. (E) Net Charge distribution of S-to-D peptides.

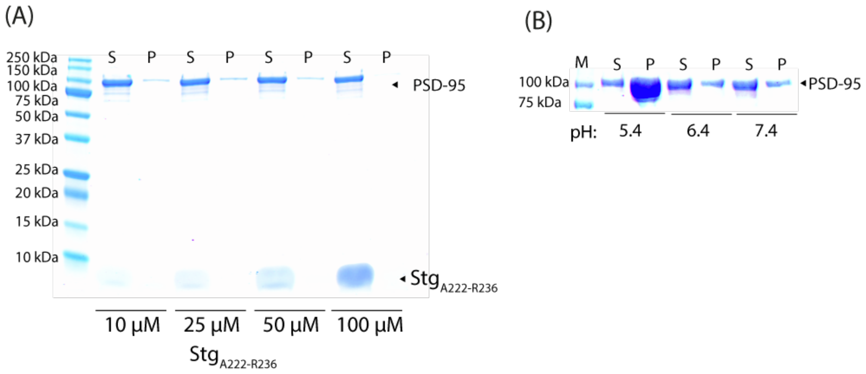

Figure S10 Representative SDS-PAGE sedimentation assay of PSD-95 incubated with StgA222-R236, related to figure 5 (A) Representative SDS-PAGE sedimentation assay of PSD-95 incubated with StgA222-R236. For quantification see Figure 4C. (B) Representative SDS-PAGE sedimentation assay of PSD-95 incubated with 50 µM StgA222-R236 at different pH values.

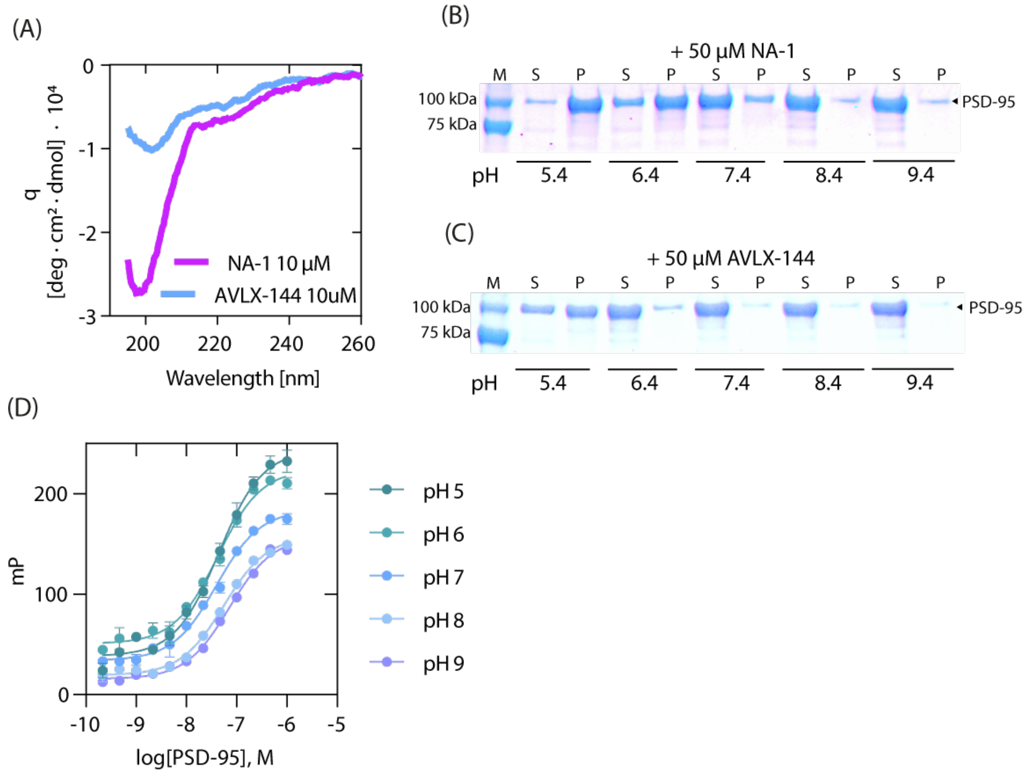

Figure S11, Solution structure and binding of PSD-95 inhibitory peptides at different pH values, related to figure 6 (A) CD spectra of 10 µM NA-1 and AVLX-144 show that both are unstructured random coils. (B) Representative SDS-PAGE gel of 3 µM PSD-95 incubated with 50 µM NA-1. (C) Representative SDS-PAGE gel of 3 µM PSD-95 incubated with 50 µM AVLX-144. (D) FP saturation binding of 5 nM AB-143 (Bach et al., 2012) towards PSD-95 at different pH values, show no major change in KD, and changes in mP values are here accredited to change in fluorescent signal due to pH changes. Error bars indicate SEM of n=3.

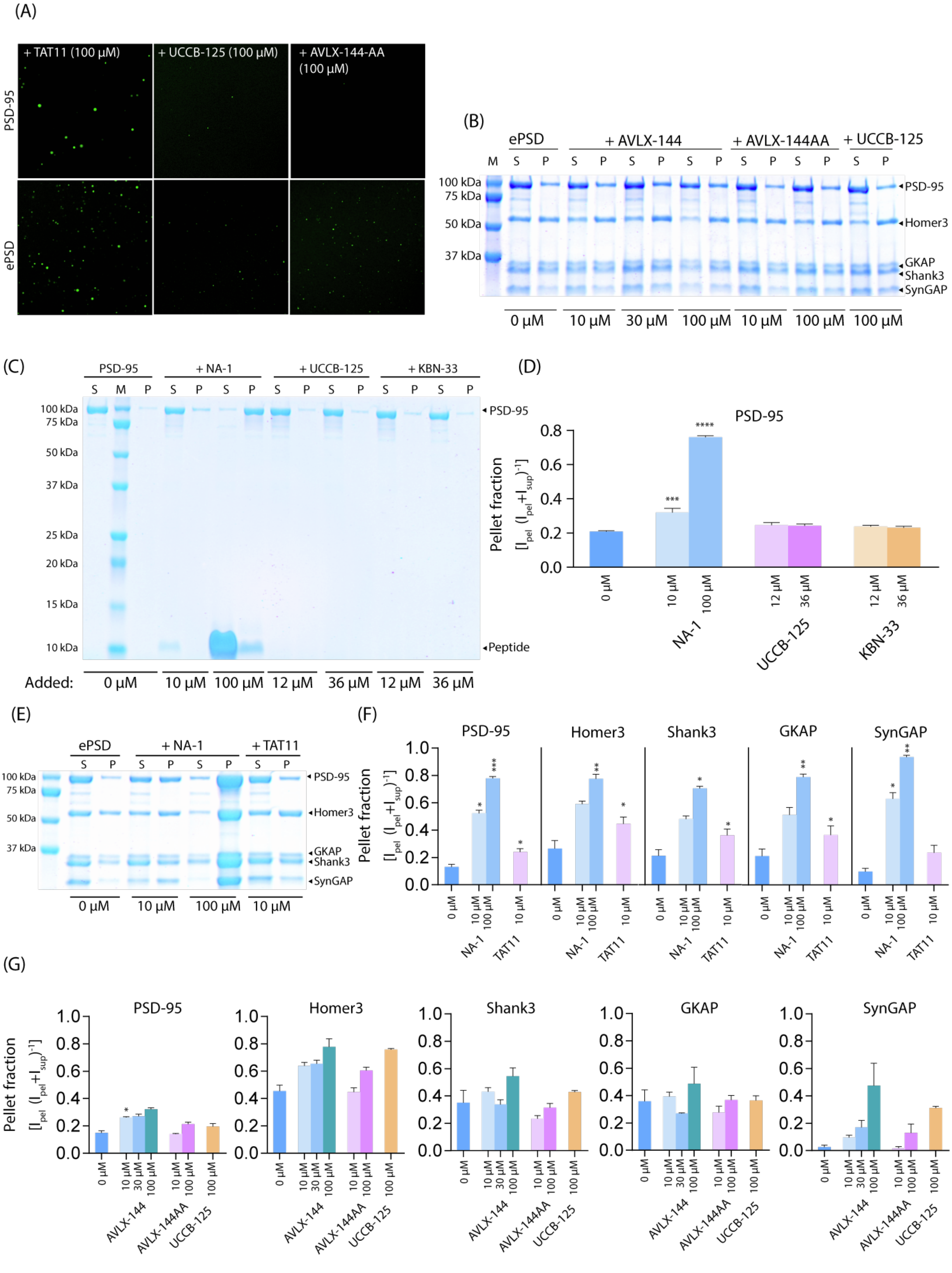
(A)
+ TAT11 (100 µM)
+ UCCB-125 (100 µM)
+ AVLX-144-AA (100 µM)
PSD-95
ePSD
(B)
ePSD
+ AVLX-144
+ AVLX-144AA
+ UCCB-125
M S P S P S P S P S P S P S P
100 kDa
75 kDa
50 kDa
37 kDa
PSD-95
Homer3
GKAP
Shank3
SynGAP
0 µM 10 µM 30 µM 100 µM 10 µM 100 µM 100 µM
(C)
PSD-95
+ NA-1
+ UCCB-125
+ KBN-33
S M P S P S P S P S P S P S P
100 kDa
75 kDa
50 kDa
37 kDa
25 kDa
20 kDa
15 kDa
10 kDa
PSD-95
Peptide
Added: 0 µM 10 µM 100 µM 12 µM 36 µM 12 µM 36 µM
(D)
PSD-95
Pellet fraction
[I_pel (I_pel+I_sup)^-1]
0.8
0.6
0.4
0.2
0.0
0 µM
10 µM
100 µM
12 µM
36 µM
12 µM
36 µM
NA-1
UCCB-125
KBN-33
(E)
ePSD
+ NA-1
+ TAT11
S P S P S P S P
100 kDa
75 kDa
50 kDa
37 kDa
PSD-95
Homer3
GKAP
Shank3
SynGAP
0 µM 10 µM 100 µM 10 µM
(F)
PSD-95
Homer3
Shank3
GKAP
SynGAP
Pellet fraction
[I_pel (I_pel+I_sup)^-1]
1.0
0.8
0.6
0.4
0.2
0.0
0 µM
10 µM
100 µM
10 µM
NA-1
TAT11
(G)
PSD-95
Homer3
Shank3
GKAP
SynGAP
Pellet fraction
[I_pel (I_pel+I_sup)^-1]
1.0
0.8
0.6
0.4
0.2
0.0
0 µM
10 µM
30 µM
100 µM
10 µM
100 µM
100 µM
AVLX-144
AVLX-144AA
UCCB-125

Figure S12 Representative SDS-PAGE sedimentation assay and quantification of PSD-95 incubated with inhibitory peptides, related to figure 6 (A) Representative images of LLPS droplets for PSD-95 (top) or ePSD (bottom) with indicated peptide. (B) SDS-PAGE sedimentation assay of ePSD (3 µM) upon addition of increasing amounts of indicated peptide. Quantification is shown in main Figure 4E. (C) Representative SDS-PAGE sedimentation assay of PSD-95 incubated with a selection of known inhibitors. NA-1 (Aarts et al., 2002), UCCB-125 (Bach et al., 2009), KBN33 (Nissen et al., 2015). (D) Quantification of (C), shows NA-1 being able to induce LLPS for PSD-95 alone. (E) SDS-PAGE sedimentation assay and quantification (F) of ePSD incubated with indicated peptides, NA-1 and TAT11, both was able to promote LLPS. (G) Quantification of SDS-PAGE sedimentation assay of ePSD incubated with indicated peptides, AVLX-144, AVLX-144-AA and UCCB-125. AVLX-144, AVLX-144-AA (Bach et al., 2012). Error bars are shown as SEM and n=3.
